# Tongbi Huoluo Decoction alleviates cartilage degeneration in knee osteoarthritis by inhibiting degradation of extracellular matrix

**DOI:** 10.1186/s13020-023-00802-z

**Published:** 2023-07-28

**Authors:** Weijian Chen, Weinian Liu, Tao Jiang, Lingyun Liu, Qi He, Tianye Lin, Jiayuan Zhang, Liwei Huo, Xuemeng Xu, Haibin Wang, Du Liang, Wengang Liu

**Affiliations:** 1 The Fifth Clinical College, Guangzhou University of Chinese Medicine, Guangzhou, 510095 China; 2 Guangdong Second Hospital of Traditional Chinese Medicine (Guangdong Province Engineering Technology Research Institute of Traditional Chinese Medicine), Guangzhou, 510095 China; 3 The First Clinical Medical College, Guangzhou University of Chinese Medicine, Guangzhou, 510405 Guangdong China; 4 Guangzhou Orthopedic Hospital, Guangzhou University of Chinese Medicine, Guangzhou, 510045 Guangdong China; 5 Department of Orthopedics, First Affiliated Hospital, Guangzhou University of Chinese Medicine, Guangzhou, 510405 Guangdong China

**Keywords:** Tongbi Huoluo Decoction, Knee osteoarthritis, Active components, Cartilage degeneration, Extracellular matrix

## Abstract

**Background:**

Knee osteoarthritis (KOA) is an age-related degenerative disease characterized by abrasion of articular cartilage. Tongbi Huoluo Decoction (TBHLD) has been transformed from the famous traditional Chinese medicine Duhuo Jisheng Decoction, which can effectively alleviate pain symptoms in KOA. However, the active components and mechanisms of TBHLD in treating KOA have not yet been elucidated. The purpose of the study was to demonstrate the molecular mechanism of TBHLD in treating KOA.

**Methods:**

The components and targets of TBHLD and KOA were collected from multiple databases, and the protein to protein interaction (PPI) network was constructed. Next, we performed topological calculation and enrichment analysis. Besides, we performed virtual screening for molecular docking and molecular dynamics simulation (MDS). Furthermore, the vitro and vivo experiments were performed to evaluate the validity and mechanism of TBHLD.

**Results:**

206 active components and 187 potential targets were screened from Tongbi Huoluo Decoction. A total of 50 intersecting genes were identified between TBHLD and KOA, 20 core targets were calculated by network topology analysis. The core targets were enriched in the ECM interaction pathways. The results of virtual screening for molecular docking and MDS showed that the active components of TBHLD had steady binding conformations with core genes. Moreover, we identified 32 differential serum components in TBHLD-containing serum using LC–MS, including 22 upregulated and 10 downregulated serum components. TBHLD improved the proliferation activity of OA chondrocytes, decreased the expression of Col1a1, Col1a2, Mmp2, Mmp13 in OA chondrocytes, ameliorated the cartilage lesions and restored the cartilage abrasion.

**Conclusion:**

TBHLD inhibited degradation of cartilage ECM by regulating the expression of type I collagens and Mmps to ameliorate cartilage degeneration in KOA.

**Supplementary Information:**

The online version contains supplementary material available at 10.1186/s13020-023-00802-z.

## Introduction

Osteoarthritis (OA) is an age-related degenerative disease with joint pain as the primary clinical symptom [1]. As the global population aging, the incidence of OA is rising every year. According to epidemiology, there are 250 million people living with osteoarthritis worldwide [2]. Epidemiological data [3] showed that 8.1% of Chinese people have knee osteoarthritis (KOA), with more than 120 million people suffering from KOA pain and limited knee movement. The characteristic pathological of KOA include deterioration of cartilage, changes of subchondral bone, osteophytes at the joint edge, and synovial inflammation [4, 5]. And the cartilage degeneration plays a critical role in the occurrence of KOA [6].

Tongbi Huoluo Decoction (TBHLD) is a popular TCM prescription, which has been transformed from Duhuo Jisheng Decoction. TBHLD is composed of thirteen herbs, including Du-Huo (DH), Xu-Duan (XD), Chuan-Wu (CW), Di-Huang (DH), Sang-Ji sheng (SJS), Dan-Shen (DS), Huang-Qi (HQ), Xi-Xin (XX), Niu-Xi (NX), Di-long (DL), Tu-Bie chong (TBC), Wu-Yao (WY), Gan-Cao (GC). TBHLD is currently predominantly utilized in the treatment of orthopedic diseases, including osteoarthritis and rheumatoid arthritis. It has shown significant efficacy in alleviating pain and discomfort associated with KOA, improving joint mobility, and protecting against structural changes in the knee joints [7, 8]. Modern pharmacological studies demonstrated that its components have effect of anti-inflammatory, analgesic, regulating cell proliferation, differentiation, and antioxidant [9, 10]. However, its bioactive components and mechanisms in treating KOA have not yet been elucidated.

**Fig. 1:**
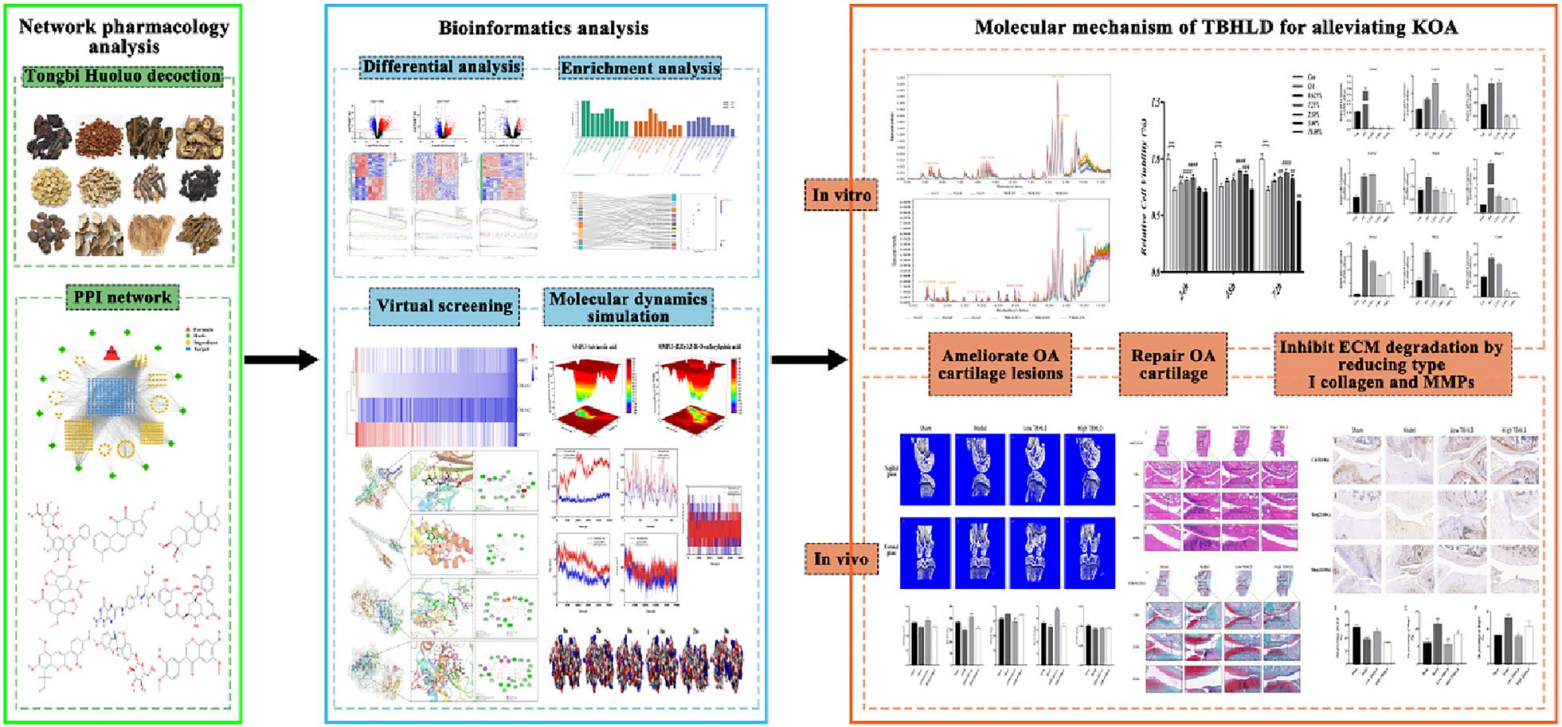
Summary of the molecular mechanism of alleviating KOA effect of TBHLD. TBHLD alleviates KOA by reducing the expression of Type I collagens and inhibiting ECM degradation

In this study, network pharmacology and bioinformatics analysis were performed to identify the active components, targets and differentially expressed genes (DEGs). The core targets and potential molecular mechanisms has been explored by applying network topology and enrichment analysis. In addition, we constructed in vitro OA chondrocytes and in vivo OA animal models to confirm the efficacy and mechanism of TBHLD in treating KOA cartilage degeneration. Finally, virtual screening for molecular docking and MDS had been carried out to identify the active components and core genes of TBHLD in treating KOA. The flow chart is shown in Fig. 1.

## Materials and methods

### Preparation of TBHLD

The compositions of TBHLD were purchased from ZISUN CHINESE PHARMACEUTICAL CO, LTD. Detailed descriptions of the herbs in TBHLD are provided in Table 1. After weighing the respective herbs according to the dosage in Table 1, they were first soaked in distilled water for half an hour and next, transferred to a reflux machine with eightfold dilution twice, and the mixture was filtered and concentrated to 2.54 g/ml and subsequently used as a crude drug.

**Table 1:** Herbs of Tongbi Huoluo Decoction

Herbs	Full taxonomy name	Latin name	Use part	Weight(g)	Batch number
Du-Huo	*Angelica dahurica* (Hoffm.)	Radix Angelicae Pubescentis	root	10	210601
Xu-Duan	*Dipsacus asper* Wall. ex DC	Radix Dipsaci	root	10	211101
Chuan-Wu	*Aconitum carmichaelii* Debeaux	Radix Aconiti	root	10	210701
Di-Huang	*Rehmannia glutinosa* (Gaertn.) DC	Radix Rehmanniae Preparata	steamed and sundried root	15	211101
Sang-Jisheng	*Taxillus chinensis* (DC.) Danser	Herba Taxilli	stem and branch-leaf	30	2111019
Dan-Shen	*Salvia miltiorrhiza* Bunge	Radix Salviae liguliobae	root	30	211201
Huang-Qi	*Astragalus mongholicus* Bunge	Radix Astragali	root	30	211201
Xi-Xin	*Asarum heterotropoides* F.Schmidt	Herba Asari	whole herb	6	210701
Niu-Xi	*Achyranthes bidentata* Blume	Radix Achyranthis bidentatae	root	10	210201
Di-Long	Earthworm	Pheretima	whole	10	210901
Tu-Biechong	Cockroach	Eupolyphaga seu Steleophaga	whole	6	T0372111
Wu-Yao	*Lindera aggregata* (Sims) Kosterm	Radix Linderae	tuberoid	10	W2122911
Gan-Cao	*Glycyrrhiza glabra L*	licorice	root and rhizome	6	211101

### Screening of TBHLD bioactive components and predicted targets

The bioactive components and related targets were collected from the Traditional Chinese Medicine Systems Pharmacology (TCMSP) database (https://tcmsp-e.com/tcmsp.php, 2022/07/15) with the screening criteria: oral bioavailability (OB) ≥ 30% [11] and drug-likeness (DL) ≥ 0.18 [12]. The bioactive compounds of Di-long (DL) and Tu-Bie chong (TBC) were obtained through literature and screened by the SwissADME platform (http://www.swissadme.ch/, 2022/12/08) based on high GI absorption and DL ≥ 2 yes [13], and their targets were predicted by the SwissTargetPrediction platform (http://www.swisstargetprediction.ch/index.php, 2022/08/08). We constructed the TBHLD-active components-targets interactions network by using Cytoscape 3.7.2 software [14].

### Differential analysis of KOA cartilage degeneration

To acquire the transcription profile data related to KOA, the GEO database (https://www.ncbi.nlm.nih.gov/geo/, 2022/12/06) was searched using the keywords "knee osteoarthritis", "cartilage degeneration", and "human". In the end, three datasets were obtained, including GSE169077, GSE113825 and GSE114007. Detailed information about the patients in the datasets is presented in Table 2. Limma. R was used to perform background correction, standardization, and differential analysis to identify the DEGs. The screening criteria are as follows: *p* < 0.05, and the expression changes were ≥ 2.00 (|log_2_ FC|≥ 1.00).

**Table 2:** Characteristics of study subjects

GEO DataSets	Group	Number of subjects	Age (years, mean ± SD)	Sex		K-L stage
				Male	Female	
GSE113825	Con	5	28.8 ± 8.23	3	2	0(n = 5)
	KOA	5	63.00 ± 3.58	2	3	IV(n = 5)
GSE114007	Con	18	36.61 ± 13.08	13	5	I(n = 18)
	KOA	20	66.20 ± 7.16	8	12	IV(n = 20)
GSE169077	Con	5	–	–	–	0(n = 5)
	KOA	6	–	–	–	IV(n = 6)

### Construction of the PPI network

Intersecting targets between TBHLD genes and KOA-DEGs were obtained by employing VennDiagram R. In addition, the PPI network of TBHLD targets and KOA–DEGs were constructed by BisoGenet [15]. CytoNCA [16] was applied for multi-centre network topology calculations of the intersected PPI between TBHLD targets and KOA–DEGs, and the medians of Degree, Betweenness Centrality (BC), Closeness Centrality (CC), and LAC were used to filter the potential core genes of TBHLD for KOA cartilage degeneration. Then, the top 20 core genes were identified by the Degree value [17].

### GO/KEGG enrichment analyses

To further explore the mechanism of core targets of TBHLD in alleviating cartilage degeneration in KOA, we performed gene ontology (GO) and Kyoto Encyclopedia of Genes and Genomes (KEGG) enrichment analysis by Bioconductor. R and ClusterProfiler. R. The top 20 gene ontology and pathways were used for visualization.

### Ethics and animals

Twenty-one 6-week-old male SD rats were employed to acquire drug-containing sera and extract primary chondrocytes, which were randomly divided into the normal serum group (n = 6), TBHLD-containing serum group (n = 6). In the TBHLD group, rats were treated with TBHLD (19.215 g/kg) twice a day for 7 consecutive days. The rats in the normal serum group received the same dose of normal saline via oral gavage. The remaining 9 rats were used for primary chondrocyte extraction. Forty 4-week-old male SD rats were used for animal experiments, and randomly divided into the sham (n = 10), model (n = 10), low-concentration (n = 10) and high-concentration (n = 10) TBHLD groups. The experiments were permitted by the Ethical Committee for Animal Research of Guangzhou University of Chinese Medicine (Certificate: No.20211105004, No.20220116001).

### Identification of serum components in TBHLD

After continuous gastric lavage for 7 days, arterial blood samples were collected from the normal serum group and the TBHLD-containing serum group. The samples were centrifuged at 4 ℃ and 3000 rpm for 15 min to obtain serum samples. An appropriate amount of sample was mixed with 400 µL of methanol solution. After centrifugation at 12,000 rpm and 4 ℃ for 10 min, the supernatant was transferred to a centrifuge tube and concentrated until dry. Then, 150 µL of a 2-chloro-L-phenylalanine (4 ppm) solution prepared in 80% methanol water was added to reconstitute the sample. The supernatant was filtered through a 0.22 μm membrane and transferred to a detection vial for LC–MS analysis. Component identification was carried out using the Thermo Vanquish ultra-high performance liquid chromatography system coupled with the Thermo Orbitrap Exploris 120 mass spectrometer. The XCMS.R package was used for peak detection, filtering, and alignment of the raw data. The identified components were further searched and compared in databases such as HMDB, massbank, LipidMaps, mzcloud, and KEGG to achieve secondary qualitative identification. Differential analysis was performed on the identified components, with a significance level set at *p* < 0.05 and VIP > 1.00, to obtain the differentially expressed serum components of TBHLD.

### Primary chondrocyte culture and cell activity assay

The cartilage tissues were removed from the surface of knee articular, and two-step digestion was carried out by adding trypsin and type II collagenase. Then, the primary chondrocytes were transferred into a CO_2_ cell incubator with DMEM/F12 complete medium. When cell confluency reached 80%, cell passaging was performed, and some chondrocytes were used to construct OA chondrocyte models by intervening with 10 ng/ml IL-1β. The activity of chondrocytes was measured by CCK8 method after incubation for 24, 48 and 72 h with various concentrations of TBHLD containing serum (0.625%, 1.25%, 2.5%, 5.0%, 10.0%).

### Quantitative real-time PCR

The optimum concentration of the serum containing of TBHLD was determined based on the CCK8 results, and the OA chondrocytes were processed with low, medium and high concentrations of TBHLD for 48 h. They were classified into normal chondrocytes, OA chondrocytes, low, medium and high-concentrations TBHLD. Total RNA was extracted and reverse-transcribed into cDNA. The amplification procedure was as follows: 50 ℃ for 2 min, 95 ℃ for 10 min, 95 ℃ for 30 s, 60 ℃ for 30 s and 40 cycles. Gapdh was used for endogenous control, and the relative expression was calculated by the 2^−ΔΔCT^ value. The primer sequences are listed in Additional file 1: Table S1.

### Establishment of OA animal model and drug administration

Forty male SD rats were randomly divided into the sham, model, low-concentration and high-concentration TBHLD groups. The model, low-concentration and high-concentration TBHLD group were constructed as OA rat models by modified Hulth method [18]: removed the anterior 1/3 of the medial meniscus and transected the anterior cruciate ligament. In the sham group, the articular cavity was opened similarly, and only saline was used for flushing the cavity. The low-concentration TBHLD was administered by gavage at 19.215 g/kg, and the high-concentration TBHLD was administered by gavage at 38.43 g/kg based on the human-rat body surface area ratio. In the sham and model groups, equal amounts of distilled water were given by gavage for six weeks.

### Micro-CT

After executed by an overdose of sodium pentobarbital, knee articular were isolated and fixed in 4% paraformaldehyde. The regions of interest analyzed in Micro-CT encompass the cartilage layer and subchondral bone of the tibial plateau. They had been scanned using sky1172 software with the following parameters: 40 kV, 600 μA current, 9 μm scanning resolution, 0.5° rotation angle and 0.5 mm AI. 3D reconstruction was performed with NRecon and CtAn software. Bone mineral density (BMD), total porosity (Po.tot), trabecular bone volume/tissue volume (BV/TV), trabecular number (Tb.N) and trabecular thickness (Tb.Th) were measured.

### Histological and immunohistochemistry assay

The knee joints were decalcified in 10% EDTA for a month. They were embedded in paraffin wax and then sectioned at 5 μm thickness, and stained with HE and Safranin O-Fast Green respectively. The degree of cartilage destruction was scored based on Osteoarthritis Research Society International (OARSI) system [19]. In addition, the slices were restored and blocked with antigen and incubated with rabbit anti-mouse CD31 (TA6191) antibody and rabbit anti-mouse Col II (TA0135), Mmp2 (TA5330), and Mmp13 (TA5355) antibody, which were obtained from Abmart Medical Technology (Shanghai) Co., Ltd. Subsequently, the second antibody labelled with horseradish peroxidase (HRP) (M212115) was added and incubated for 30 min. Finally, slices were stained with the DAB kit, and visualized the cartilage layer of the medial and lateral condyles of the femur and the tibial plateau under a microscope.

### Virtual molecular docking

The active components of TBHLD were downloaded from the PubChem database. The core proteins were searched from Protein Data Bank (PDB), pre-processed with Pymol software for dehydration and dephosphorylation, then imported into Discovery Studio 2019 for batch screening. The active pocket position was set to "From PDB Site Records" and the docking radius was set to 12.00, the LibDock module performed the flexible docking. The best-docked protein-component complexes from the virtual screening results were further processed by Autodock/Vina software to complete the docking between the active components and core proteins and visualized by Pymol to construct a molecular docking pattern map.

### MDS validation

MDS, an advanced method for investigating the reliability and kinetic characteristics of complexes in aqueous solutions [20], was performed by Gromacs 2019.6 software. The amber14sb was programmed as the protein force field, and the Gaff2 force field was applied to small molecules, and the TIP3P water model was added to complex system with a sodium ion equilibrium system. The system was balanced by NVT system and NPT system, followed by a 50 ns MDS.

### Statistical analysis

All data are expressed as mean ± SD. Comparisons between groups were accomplished by ANOVAs, followed by Bonferroni’s tests for multiple comparison. The statistical calculations were carried out by SPSS Statistics 25.0, and *p* < 0.05 was considered statistically significant.

## Results

### Identification of bioactive components and targets

**Fig. 2:**
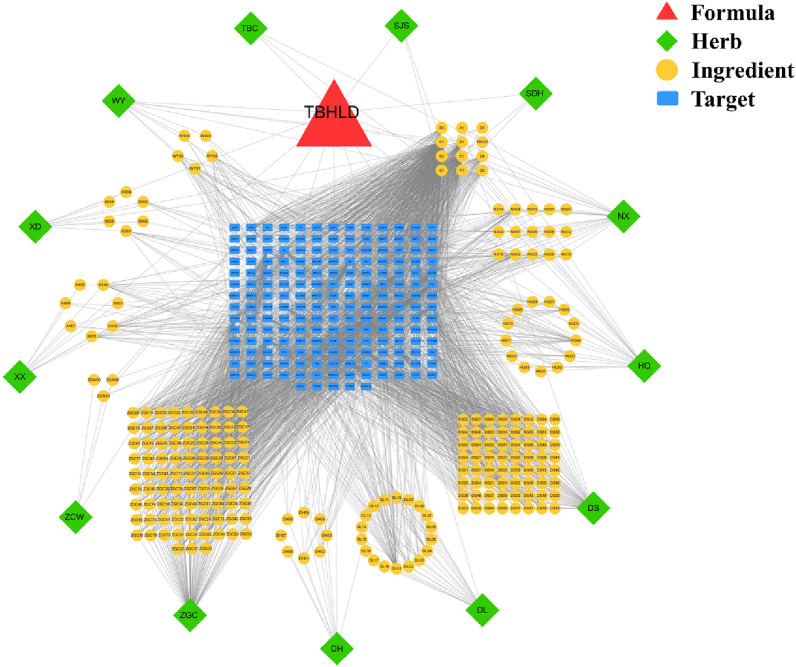
PPI network of TBHLD-herbs-compounds-targets. The red triangle represents TBHLD, the green diamond represents herbs of TBHLD, the yellow circle represents the bioactive compounds, and the blue rectangle represents the targets

A total of 206 bioactive components and 187 potential targets were identified from the TCMSP database. The PPI network of TBHLD-active components-targets interactions is shown in Fig. 2. The detailed information of the bioactive components and potential targets of TBHLD is presented in Additional file 2.

### Differential analysis of KOA

**Fig. 3:**
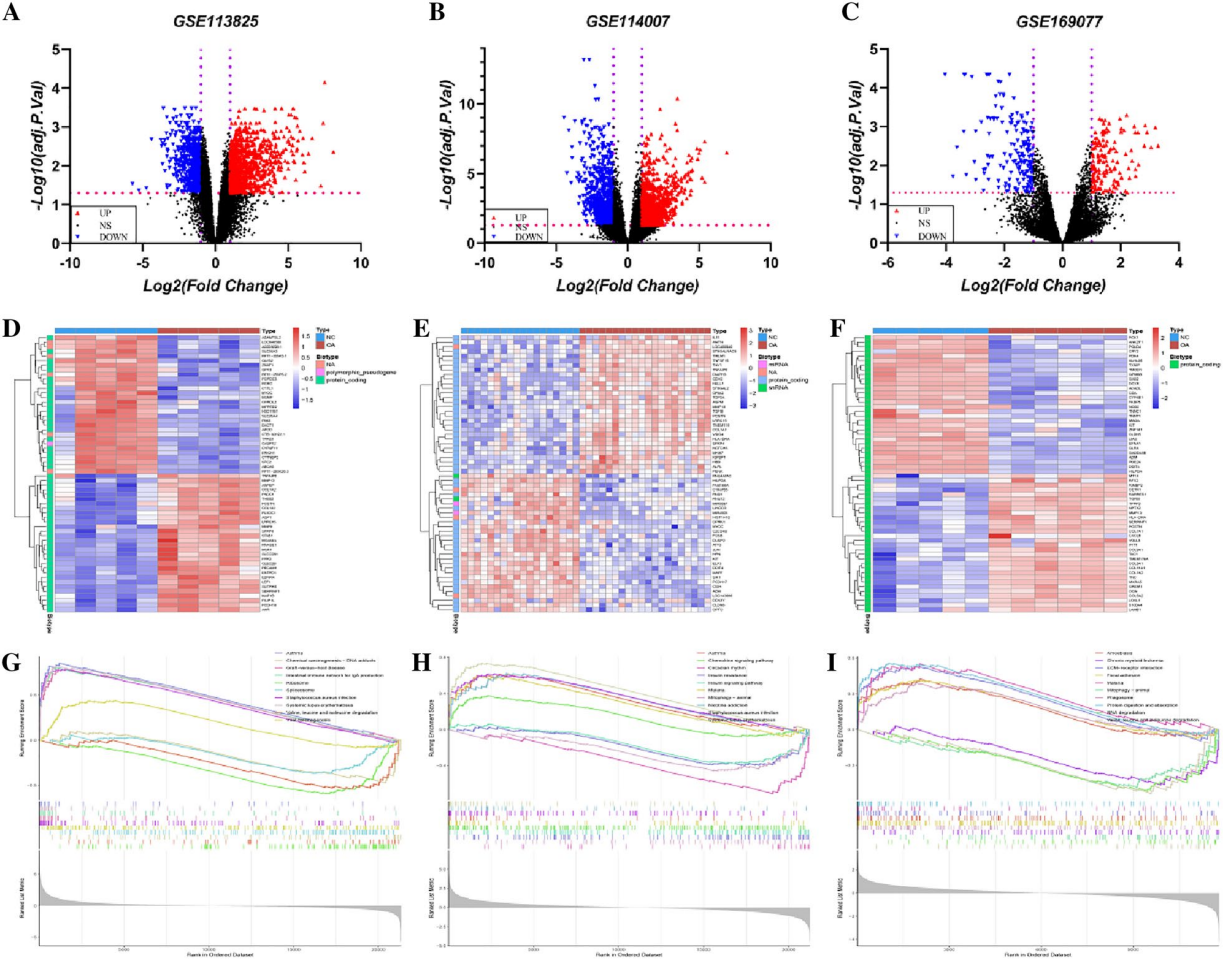
DEGs of cartilage degeneration in KOA. The volcano plot and heatmap of GSE113825 (**A**, **D**), GSE114007 (**B**, **E**), and GSE169077 (**C**, **F**). Blue, red, and black dots represent downregulated, upregulated, and unchanged genes, respectively. The GSEA enrichment of GSE113825 (**G**), GSE114007 (**H**), and GSE169077 (**I**)

1810 DEGs were identified from GSE113825, including 988 up-regulated genes and 822 down-regulated genes. 1899 DEGs were obtained in GSE114007, among which 967 DEGs were up-regulated and 932 DEGs were down-regulated. Additionally, 339 DEGs, including 147 up-DEGs, and 192 down-DEGs, were obtained in GSE169077. Volcanos and heatmaps for the three datasets are shown in Fig. 3A–F. GSEA analysis of DEGs in the above datasets revealed that they were significantly enriched in ECM-receptor interaction, focal adhesion, and mitophagy compared to normal cartilage tissue(*p* < 0.01) (Fig. 3G–I).

### The intersection genes of TBHLD in KOA

**Fig. 4:**
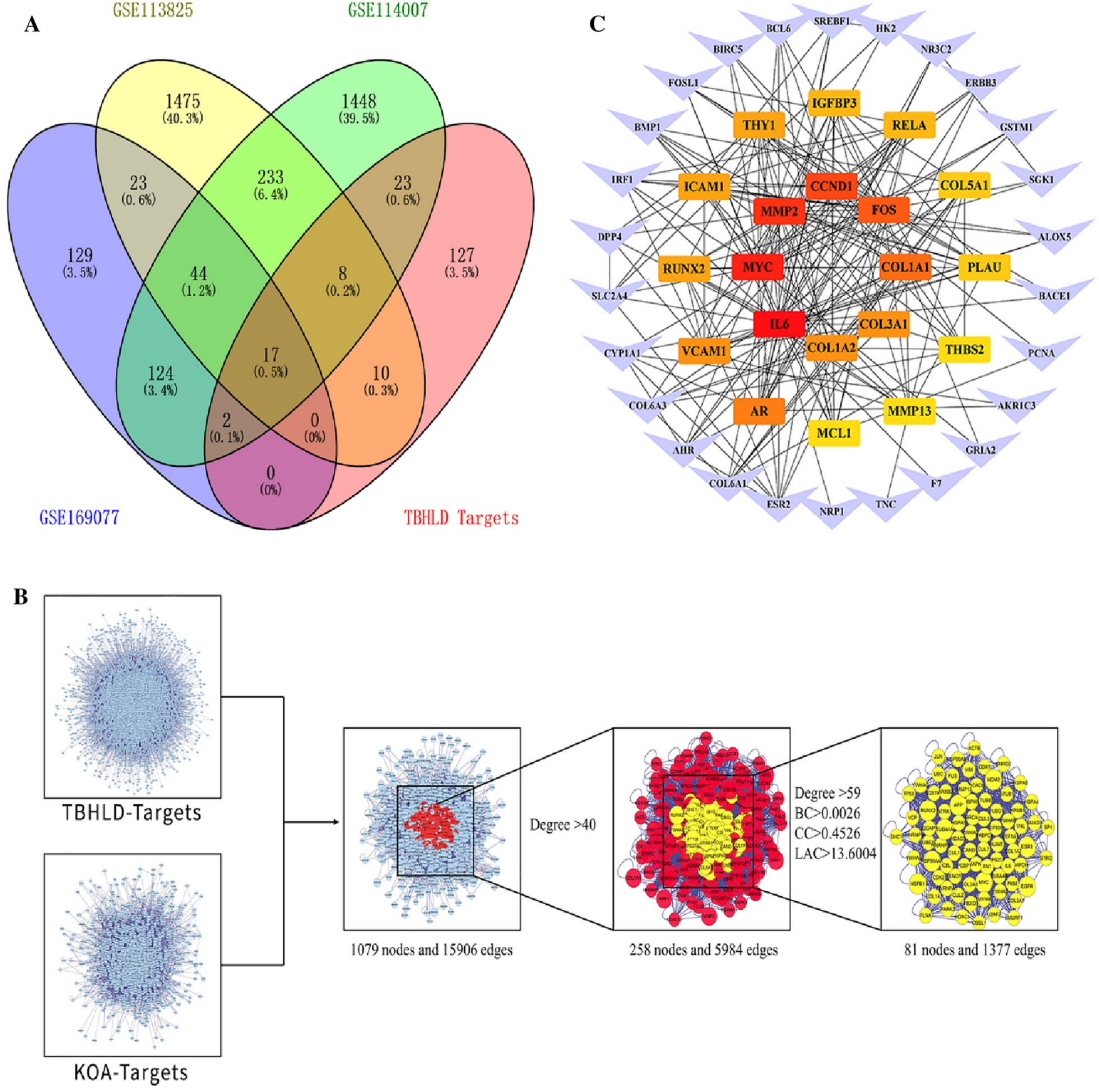
The potential core targets identified by PPI analysis. **A** Venn diagram showing the targets of TBHLD and KOA. **B** The screening process of PPI network topology. **C** The network of core targets (the red rectangles represent the core genes in the network, with darker shades indicating higher Degree values, the purple triangles represent non-core genes with Degree values < 9)

187 TBHLD targets were matched with KOA-DEGs to obtain 50 potential genes for treating cartilage degeneration in KOA with TBHLD. The venn diagram of TBHLD-KOA intersection targets is shown in Fig. 4A. Meanwhile, the active components-targets and KOA-DEGs PPI networks were constructed by BisoGenet, and the above networks were merged to create the intersection network. The results showed 258 nodes and 5984 edges in the secondary network, based on Degree > 40 (two times the median of Degree). Further filtering with Degree > 59, BC > 0.0026, CC > 0.4526 and LAC > 13.6004, the result showed 81 nodes and 1377 edges in the three-level network (Fig. 4B). Finally, the top 20 core targets were identified according to the Degree value including COL1A1, COL1A2, COL3A1, COL5A1, MMP2, MMP13, RUNX2, THBS2, CCND1 (Fig. 4C).

### Enrichment analysis

**Fig. 5:**
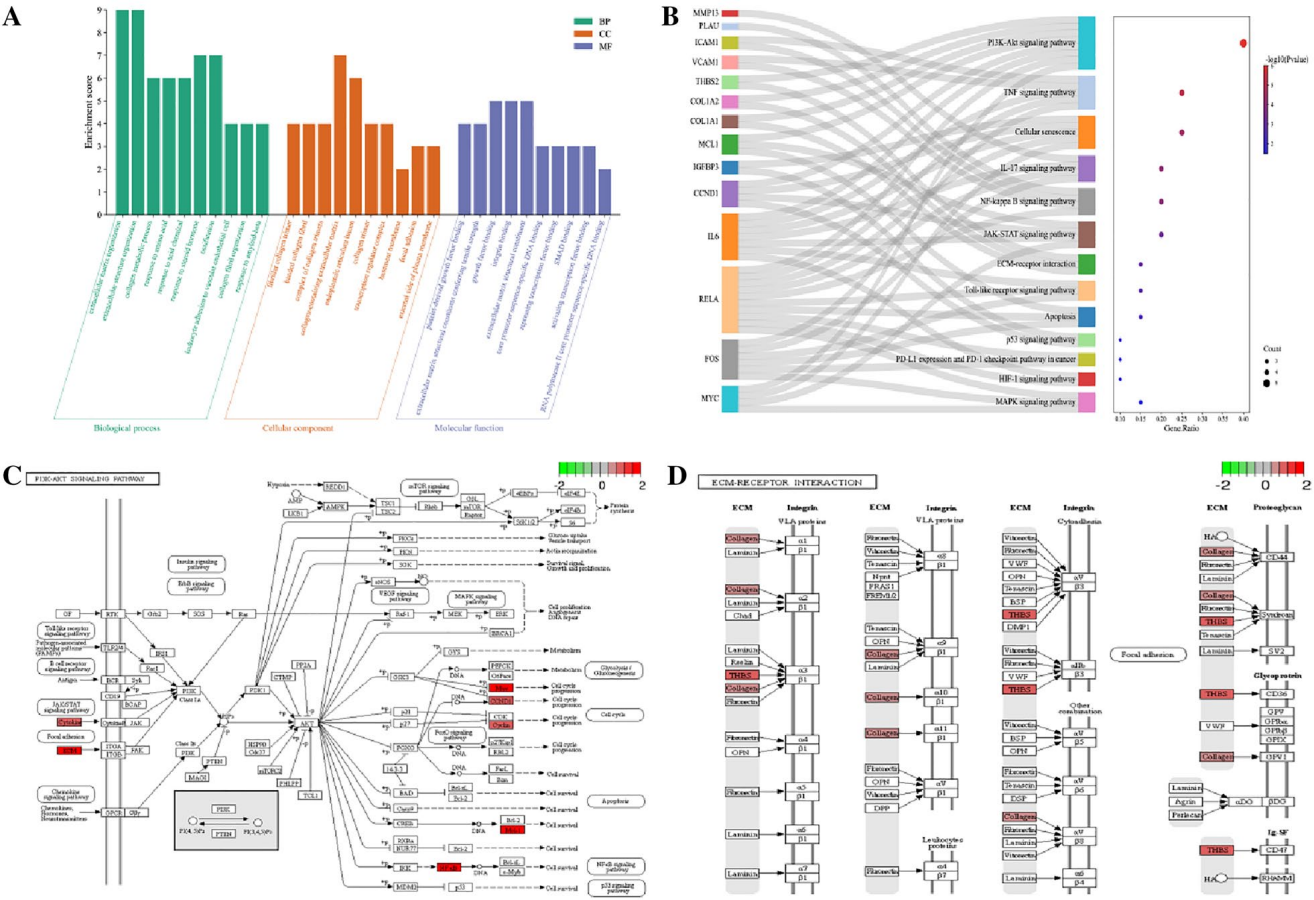
Enrichment analysis. **A** Bar chart of GO enrichment analysis. **B** Sankey diagram of the KEGG pathway. The distribution of core targets in PI3K-Akt pathway (**C**) and ECM-receptor interaction pathway **D**

Enrichment analysis of 20 core targets revealed that GO was mainly enriched in ECM organization and collagen metabolic process (Fig. 5A). KEGG was mainly enriched in ECM-receptor interaction, PI3K-Akt and TNF signaling pathway (Fig. 5B). The signaling pathways details correlated with TBHLD treatment of cartilage degeneration in KOA are shown in Fig. 5C, D.

### Identification of the serum components in TBHLD

**Fig. 6:**
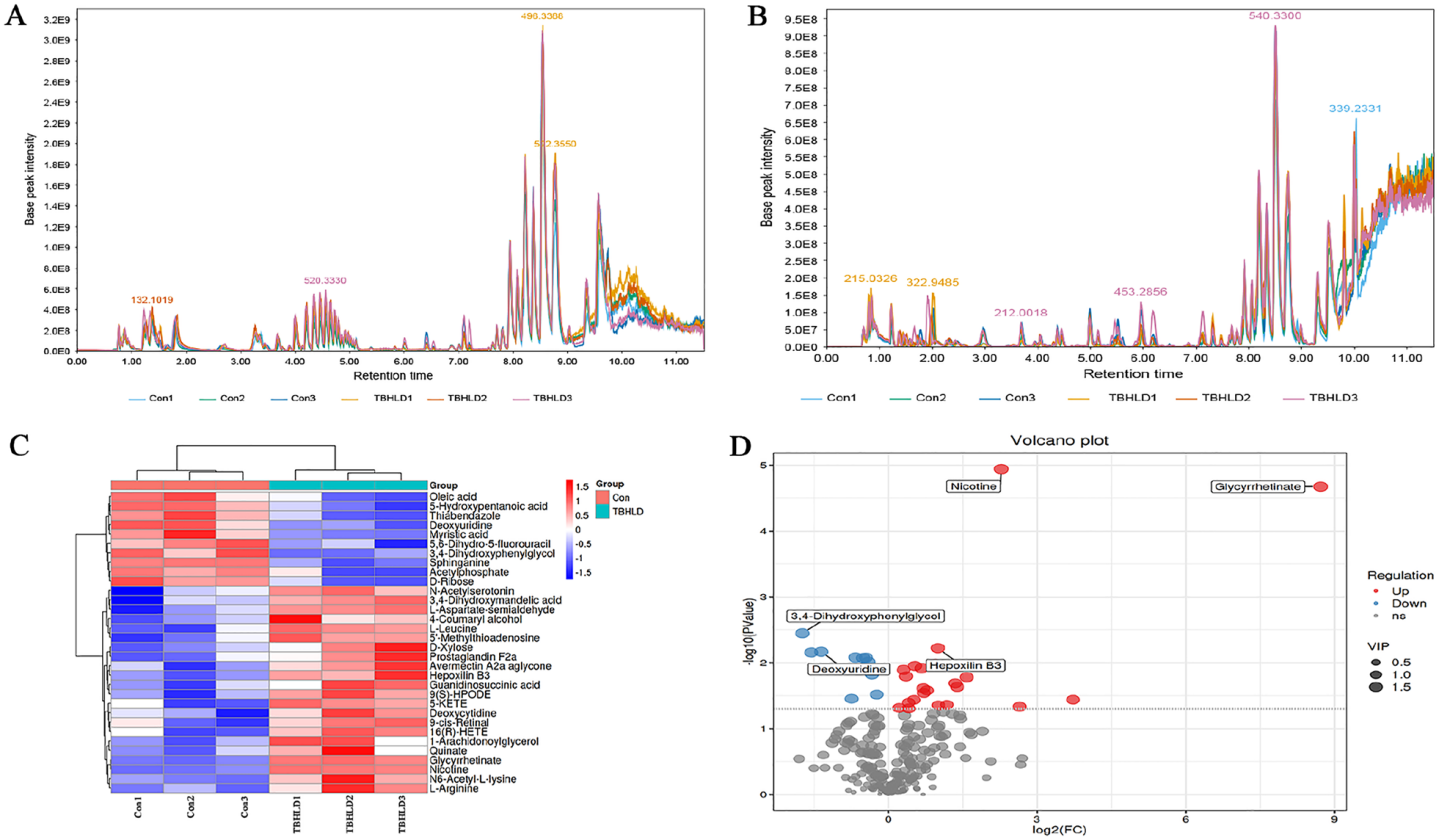
LC–MS analysis of TBHLD serum components. Chromatographic peak profiles of compounds **A** in positive ion mode; **B** in negative ion mode. Differential expression of serum components shown in **C** heatmap; **D** volcano plot

Components eluted from chromatographic separation continuously enter the mass spectrometer, and the mass spectrometer performs consecutive scans for data acquisition. Each scan generates a mass spectrum, and the ions with the highest intensity in each mass spectrum are selected for continuous plotting, with ion intensity as the y-axis and time as the x-axis, resulting in the base peak chromatogram (BPC) for each sample (Fig. 6A, B). Using information such as mass to charge ratio, retention time, and intensity of compounds in both positive and negative ion modes, combined with compound mass spectra, a total of 259 serum components were identified. Through differential analysis of serum components between the normal serum group and the TBHLD-containing serum group, 16 differentially expressed serum components were found in both positive and negative ion modes, including 22 upregulated and 10 downregulated serum components (Table 3). The heatmap and volcano plot of the differentially expressed serum components can be seen in Fig. 6C, D.

**Table 3:** LC–MS measurement parameters of differentially expressed serum components in TBHLD

No	Name	mz	rt/s	ppm	log_2_FC	p.value	VIP	Type
1	5-Hydroxypentanoic acid	119.073	201.6	22.87672	− 0.45	0.008342	1.683273	pos
2	L-Leucine	132.1019	80.8	0.181678	0.31	0.012864	1.66091	pos
3	5,6-Dihydro-5-fluorouracil	133.0318	72.2	0.006806	− 0.33	0.015022	1.624164	pos
4	Acetylphosphate	139.982	31.9	0.435395	− 0.23	0.03028	1.552004	pos
5	3,4-Dihydroxyphenylglycol	169.9773	92.7	0.452329	− 1.73	0.003585	1.728516	pos
6	3,4-Dihydroxymandelic acid	184.1695	126.5	1.390554	0.72	0.028783	1.555177	pos
7	N6-Acetyl-L-lysine	189.1234	45.6	0.000388	0.54	0.011194	1.671375	pos
8	Thiabendazole	202.044	201.4	1.113206	− 0.4	0.009698	1.678637	pos
9	Deoxyuridine	228.1958	641.8	0.06115	− 1.35	0.00684	1.698428	pos
10	Myristic acid	229.1797	412.6	1.785623	− 0.51	0.00848	1.693119	pos
11	Oleic acid	282.2787	521	0.632864	− 0.74	0.035148	1.541356	pos
12	9-cis-Retinal	285.2195	529.9	6.226783	1.01	0.044273	1.498674	pos
13	Sphinganine	302.3042	491.1	0.969481	− 0.66	0.008316	1.789165	pos
14	1-Arachidonoylglycerol	361.2723	462.4	1.648725	0.71	0.024507	1.602941	pos
15	Glycyrrhetinate	453.3405	555.5	18.38353	8.72	2.12E-05	1.818695	pos
16	Avermectin A2a aglycone	616.3658	432.8	7.6254	0.78	0.026296	1.576102	pos
17	L-Aspartate-semialdehyde	116.0352	48	1.06864	0.67	0.012096	1.65956	neg
18	4-Coumaryl alcohol	131.0501	192.5	2.975961	2.65	0.046341	1.527998	neg
19	D-Xylose	149.0093	544.6	7.012952	0.41	0.04028	1.476882	neg
20	D-Ribose	149.9939	550.4	5.387926	− 1.55	0.00701	1.664844	neg
21	Nicotine	160.9331	689.6	8.20923	2.28	1.15E-05	1.810164	neg
22	Quinate	173.0447	136.4	1.791445	0.41	0.04958	1.503161	neg
23	L-Arginine	173.1042	59.8	1.294018	0.36	0.016083	1.593975	neg
24	Guanidinosuccinic acid	174.9571	118.8	12.42855	3.73	0.036359	1.51136	neg
25	N-Acetylserotonin	199.1702	518.6	5.170116	0.22	0.048617	1.507976	neg
26	Deoxycytidine	226.0828	72.8	2.211579	0.52	0.036608	1.477014	neg
27	9(S)-HPODE	293.2118	462.9	0.306945	0.95	0.010478	1.669072	neg
28	5′-Methylthioadenosine	297.2452	554.8	6.130677	1.58	0.016614	1.653746	neg
29	5-KETE	317.2107	465.3	4.728718	1.35	0.020487	1.589074	neg
30	16(R)-HETE	319.2256	497.6	6.96686	1.39	0.023236	1.553453	neg
31	Prostaglandin F2a	335.2198	418	7.189313	1.18	0.043442	1.477773	neg
32	Hepoxilin B3	335.2206	450.3	6.562842	1	0.005992	1.673223	neg

mz: mass-to-charge ratio; rt: retention time; type: ionization mode, pos for positive ion mode, neg for negative ion mode; ppm: parts per million; VIP: variable importance in projection value of OPLS-DA first principal component

### TBHLD ameliorated OA chondrocyte lesions

**Fig. 7:**
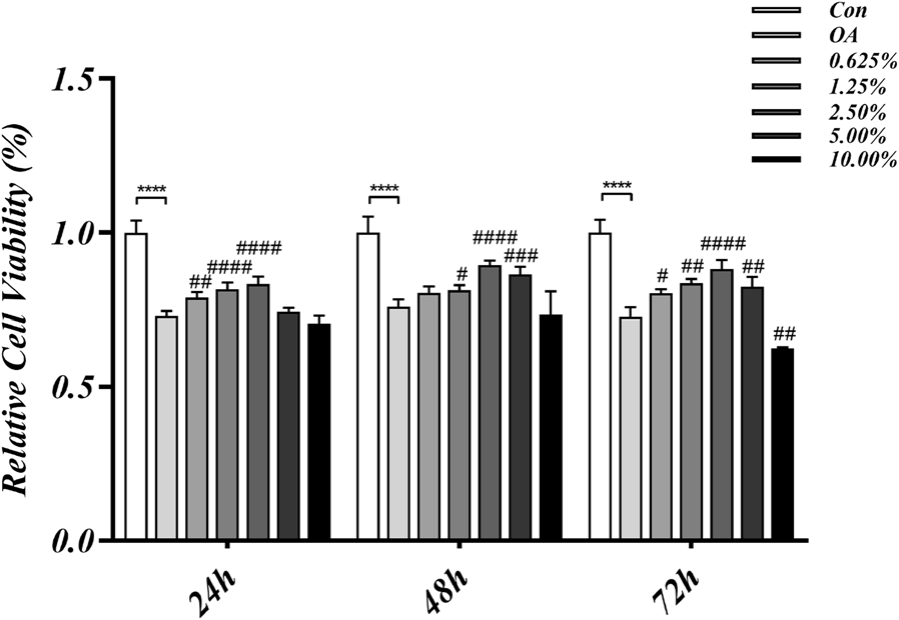
Effect of TBHLD on the viability of IL-1β-induced OA cells. ****p < 0.0001 vs. Control; ##*p* < 0.01, ###*p* < 0.001 and ####*p* < 0.0001 vs. OA

CCK8 results showed that the proliferative activity of OA chondrocytes was markedly lower than normal chondrocytes (*p* < 0.05). The concentrations of 1.25%, 2.50% and 5.00% serum ameliorated IL-1β damage in chondrocytes (*p* < 0.05) and improved chondrocyte proliferation activity. In contrast, when the concentration of TBHLD reached 10.00%, it reduced the activation of chondrocytes. The chondrocyte proliferation activity curves for each group are shown in Fig. 7, and the detailed statistical values are presented in Additional file 1: Table S2.

### TBHLD decreased type I collagen and Mmps expression

**Fig. 8:**
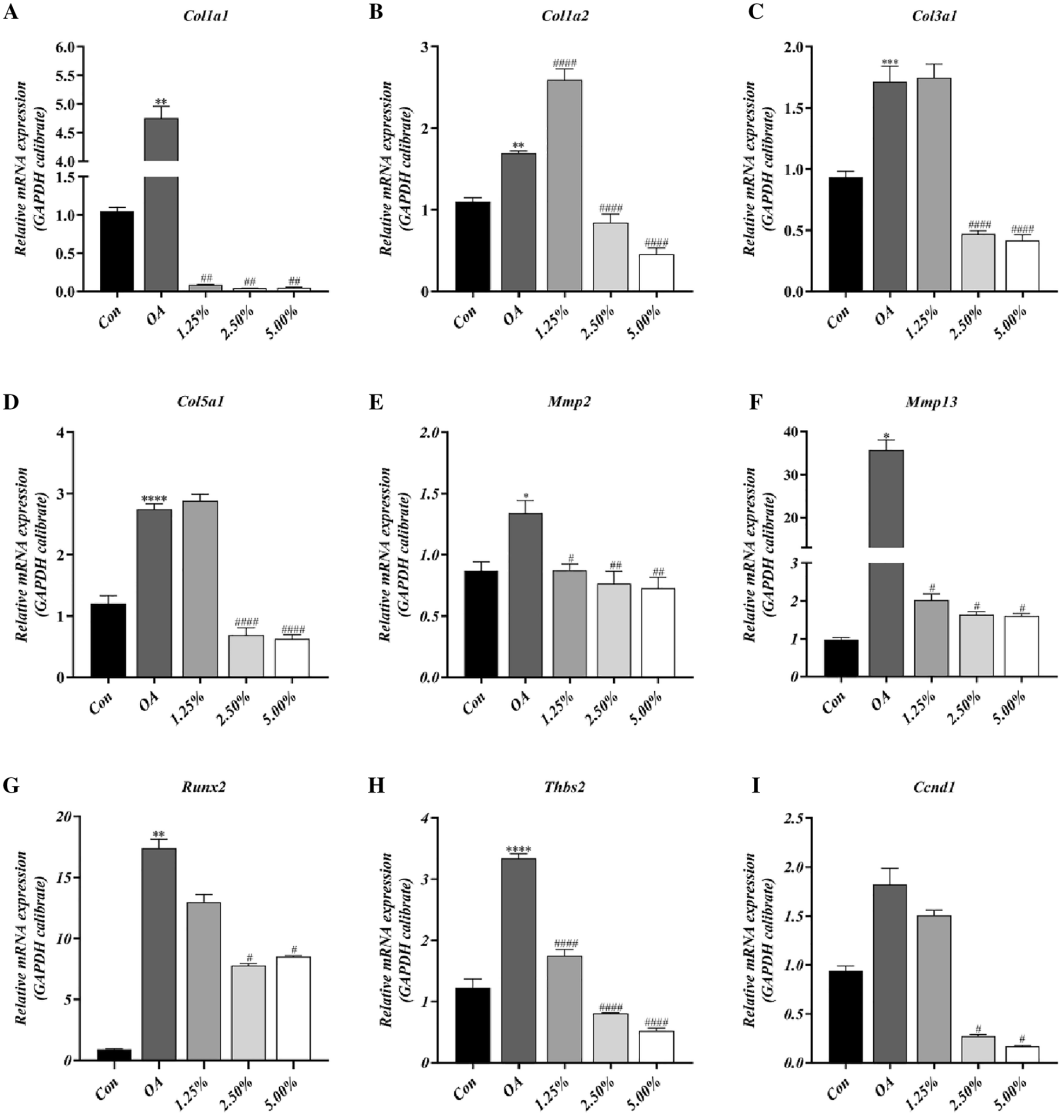
The effects of TBHLD treatment on the mRNA expression of KOA core genes. **A** Col1a1. **B** Col1a2. **C** Col3a1. **D** Col5a1. **E** Mmp2. **F** Mmp13. **G** Runx2. **H** Thbs2. **I** Ccnd1. *p < 0.05, **p < 0.01, ***p < 0.001 and****p < 0.0001 vs. Control; ^#^*p* < 0.05, ^##^*p* < 0.01 and ^####^*p* < 0.0001 vs. OA

According to the results of CCK8, 1.25%, 2.50% and 5.00% drug-containing serum were selected to intervene in OA chondrocytes, and total chondrocyte RNA was extracted for qRT-PCR quantification. The results showed that the expression of OA chondrocytes in Col1a1, Col1a2, Col3a1, Col5a1, Mmp2, Mmp13, Runx2 and Thbs2 were markedly upregulated as compared to normal chondrocytes (*p* < 0.05). The expression of Col1a1, Mmp2, Mmp13 and Thbs2 was decreased significantly in the OA group under treatment with 1.25% drug-containing serum (*p* < 0.05). The expression of Col1a1, Col1a2, Col3a1, Col5a1, Mmp2, Mmp13, Runx2, Thbs2 and Ccnd1 decreased significantly when treated with 2.50% and 5.00% drug-containing serum compared to the OA group (*p* < 0.05) (Fig. 8A–I). The statistical values are shown in Additional file 1: Table S3. Based on the results, TBHLD markedly decreased the expression of Col1a1, Col1a2, Mmp2, and Mmp13 in OA chondrocytes and ameliorated injure of OA chondrocytes.

### TBHLD alleviated cartilage lesions and subchondral bone loss

**Fig. 9:**
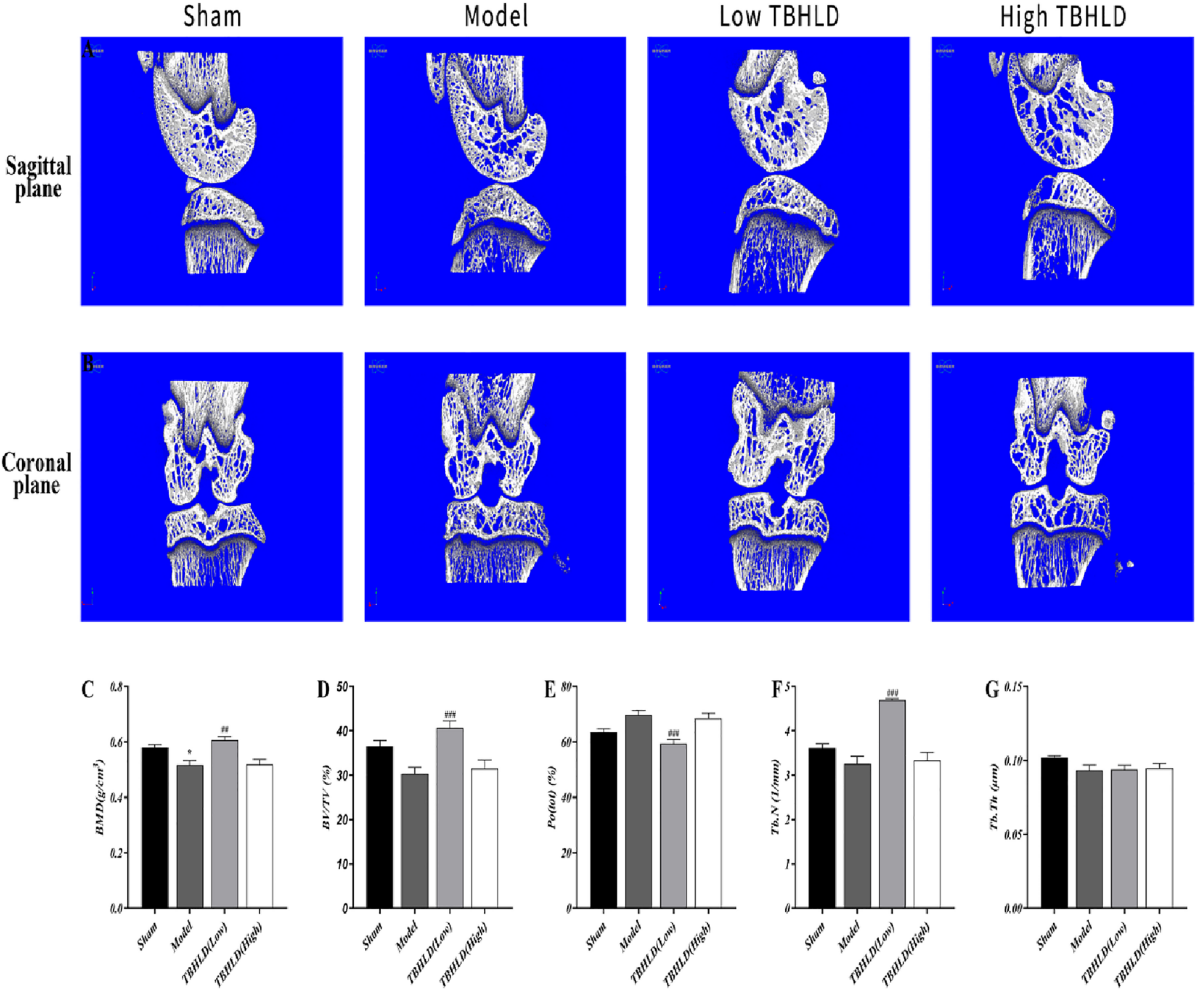
TBHLD prevented cartilage damage and bone loss in vivo. **A** Representative 3D reconstruction micro-CT images of knee joint in the different groups in the sagittal plane. **B** Representative 3D reconstruction micro-CT images of knee joint in the different groups in the coronal plane. **C**–**G** BMD, BV/TV, Po(tot), Tb. N, Tb. Th were analyzed based on micro-CT. *p < 0.05 vs. Sham; ^##^*p* < 0.01 and ^###^*p* < 0.001 vs. Model

The microstructural changes of the cartilage layer, subchondral bone of the tibial plateau and distal femur were subsequently examined by micro-CT to clarify the effect of TBHLD on the cartilage of KOA rats (Fig. 9A, B). Compared to the sham group, BMD was markedly lower in the model group (*p* < 0.05). In addition, BMD, Tb.N and BV/TV increased markedly in the low TBHLD group compared to the model group, while Po(tot) decreased significantly compared to the model group (*p* < 0.05). The results indicated that low-concentration TBHLD could markedly improve the quantity of subchondral bone and alleviate the destruction of the cartilage (Fig. 9C–G). The detailed statistical values are presented in Additional file 1: Table S4.

### TBHLD promoted cartilage repair in OA

**Fig. 10:**
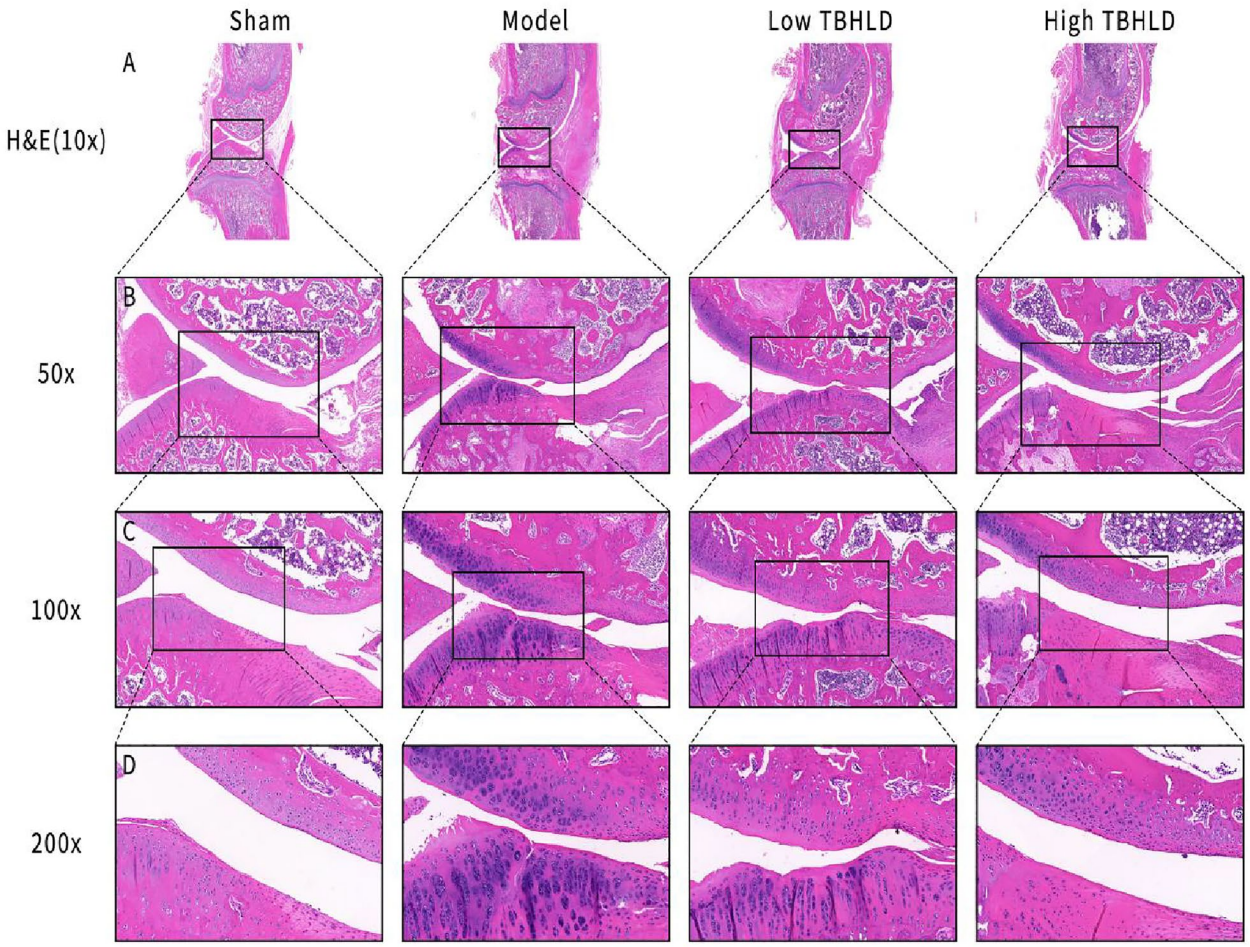
HE staining of KOA cartilage tissue. **A**–**D** Images were captured at 10X, 50X, 100X, 200X magnification in the different groups

**Fig. 11:**
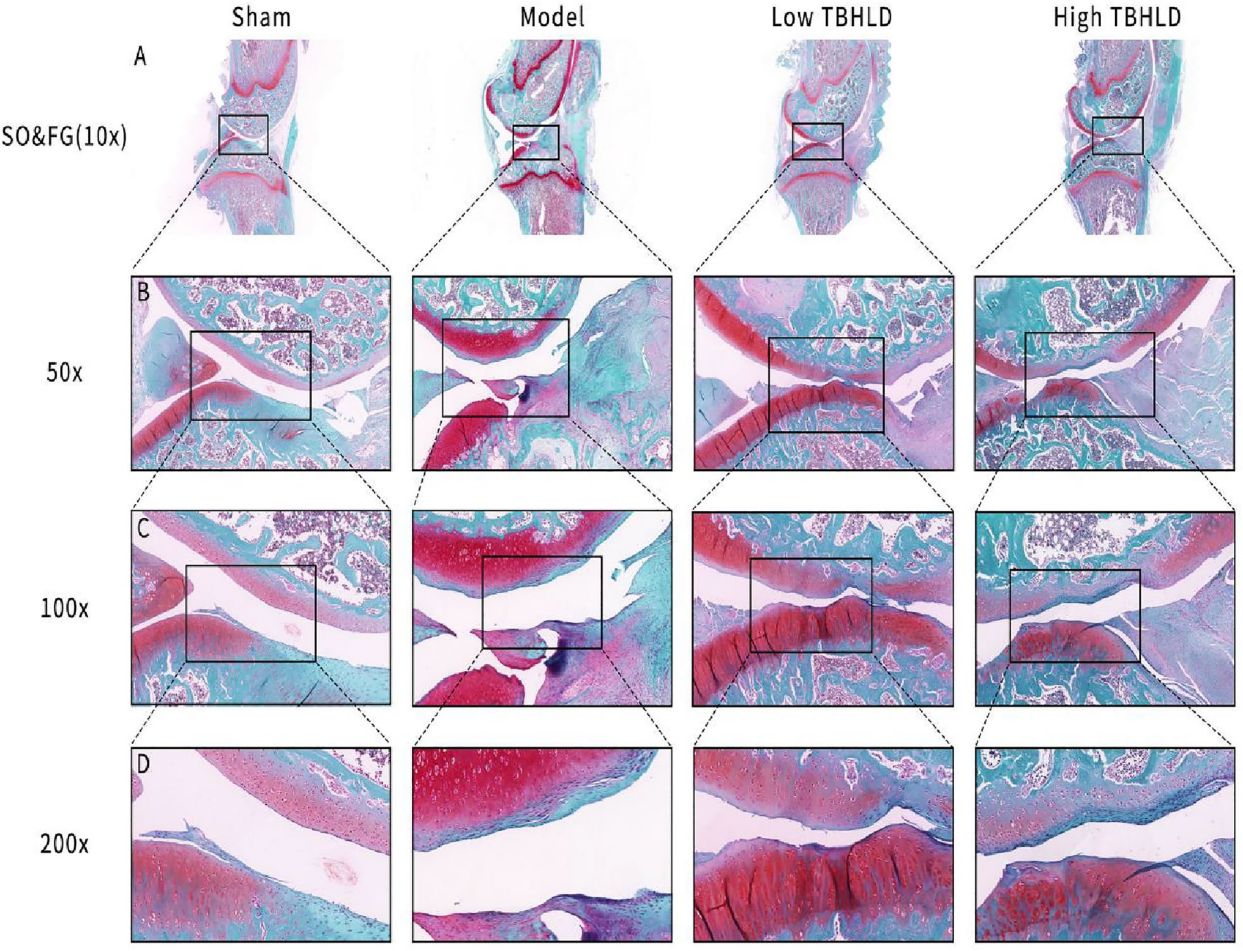
SO&FG staining of KOA rats cartilage tissue. **A**–**D** Images were captured at 10X, 50X, 100X, 200X magnification in the different groups

The HE and SO&FG staining showed that the articular cartilage surface of model group were irregular, the cartilage layers were thinned or absent, fissures were locally observed and penetrated deeply into subchondral bone layer, and the subchondral bone structure were disturbed with significant lesion defects. In the low TBHLD group, the articular cartilage surfaces were still irregular, but the layers were intact and the chondrocytes were hyperplastic. In the high TBHLD group, the cartilage lesion fissures extended to the subchondral bone layer, and partial cartilage lesion defects were present, with no apparent difference compared to the model group (Figs. 10A–D and 11A–D). In addition, the OARSI score for knee cartilage pathology is shown in Additional file 1: Table S5, which revealed that the OARSI score was markedly higher in the model group (*p* < 0.01). In contrast, the OARSI score dramatically decreased in the low and high-concentration TBHLD groups compared to the model group (*p* < 0.001).

### TBHLD decreased Mmp2/Mmp13 and enhanced collagen II expression

**Fig. 12:**
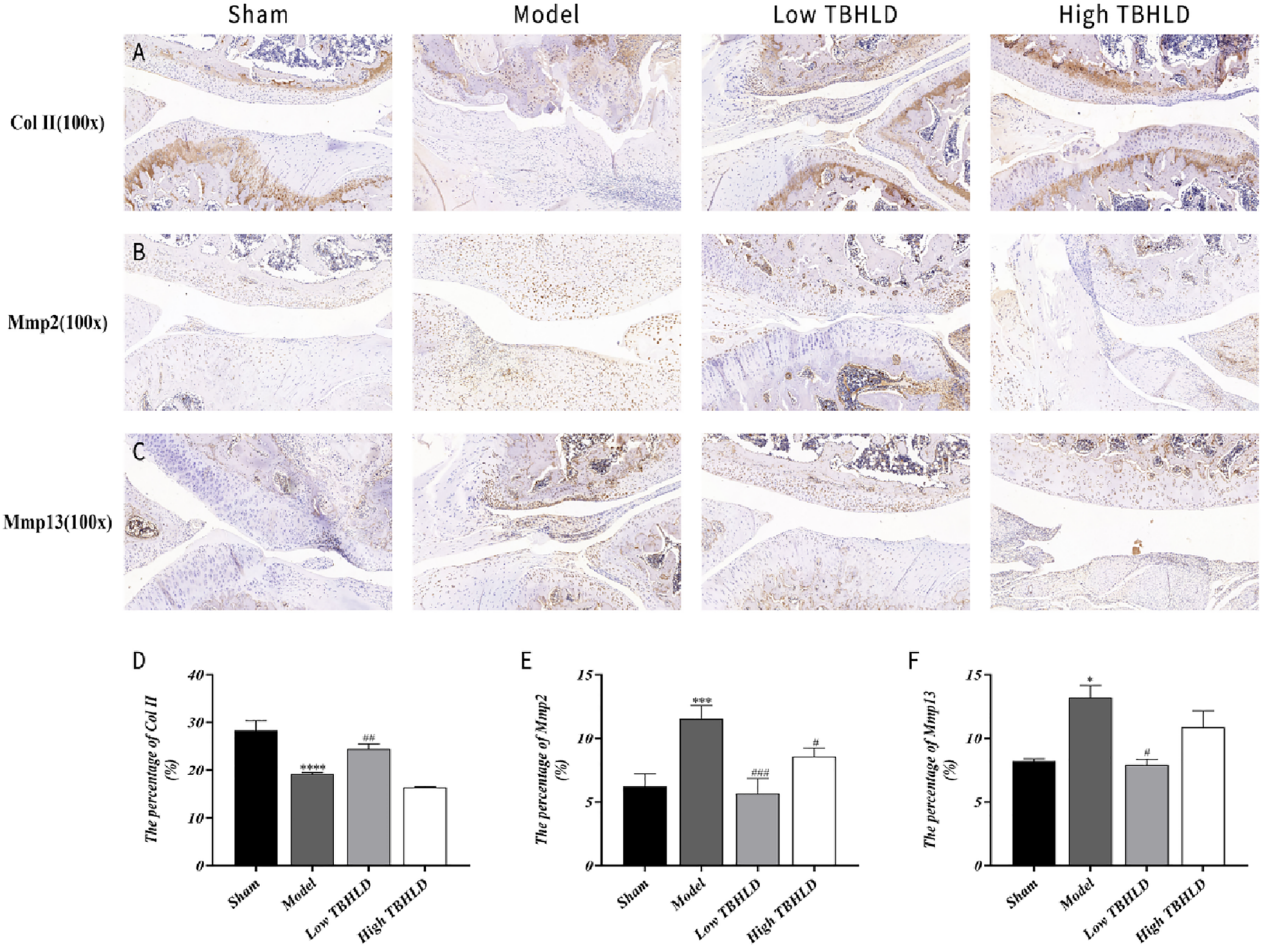
TBHLD repaired cartilage damage of KOA cartilage. Positive expression of **A** Col II, **B** Mmp2 and **C** Mmp13 protein in knee cartilage sections detected by immunohistochemical staining. Images were captured at 100X magnification. Col II **D**, Mmp2 **E** and Mmp13 **F** are quantified as positive cell rate. In each group, respectively. *p < 0.05, ***p < 0.001 and ****p < 0.0001 vs. Sham; ^#^*p* < 0.05, ^##^*p* < 0.01 and ^###^*p* < 0.001 vs. Model

IHC results revealed that the expression of Mmp2 and Mmp13 was markedly increased, and the expression of Col II protein was decreased in the model group (*p* < 0.05). The expression of Mmp2 and Mmp13 was decreased, while Col II was increased (*p* < 0.05) in the low TBHLD group as compared to the model group. Compared with the model group, the expression of Col II was further decreased in the high TBHLD group (*p* < 0.01). The above results demonstrated that low-concentration TBHLD inhibited ECM degradation in KOA (Fig. 12A–C). The relative expression of Col II and Mmp2/Mmp13 proteins are shown in Fig. 12D–F, and the statistical data is presented in Additional file 1: Table S6.

### Virtual molecular docking

**Fig. 13:**
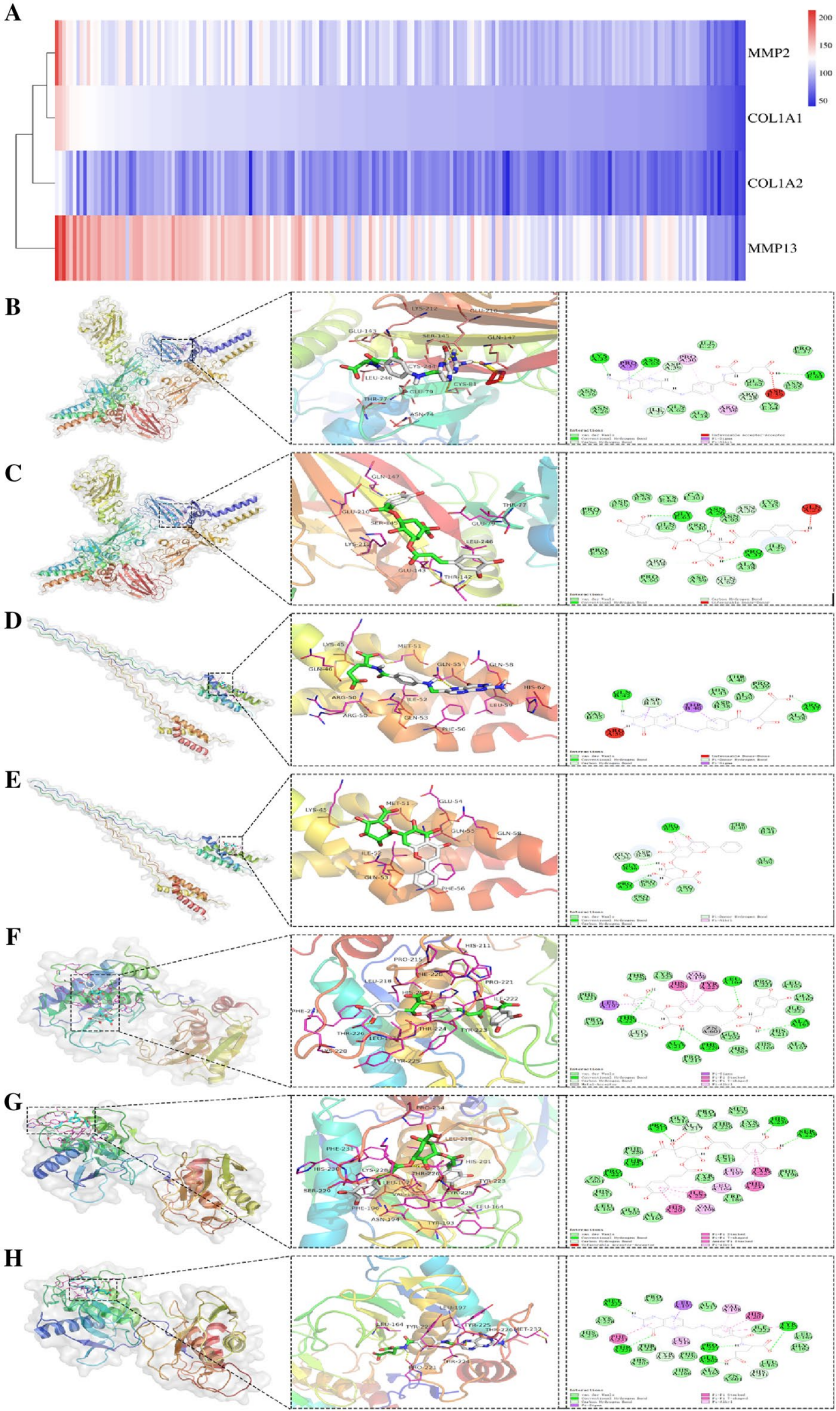
The compound-protein virtual screening and molecular docking. **A** The heatmap of molecular docking score on the hub targets (COL1A1, COL1A2, MMP2, and MMP13) and related bioactive components. The docking mode of COL1A1 with FA, (E,E)-3,5-Di-O-caffeoylquinic acid **B**, **C**, COL1A2 with FA, Baicalin **D**, **E**, and MMP13 with Salvianolic acid, (E,E)-3,5-Di-O-caffeoylquinic acid, FA **F**–**H**

The LibDockScore clustering heatmap is shown in Fig. 13A. The virtual screening results showed that COL1A1 formed the best docking with Salvianolic acid, FA and (E,E)-3,5-Di-O-caffeoylquinic acid, COL1A2 formed the best docking with FA, Baicalin and Salvianolic acid, while MMP2 formed the best docking with Salvianolic acid, FA and (-)-Medicocarpin, and MMP13 formed the best docking with Salvianolic acid, (E,E)-3,5-Di-O-caffeoylquinic acid, FA. The above protein-component complexes were selected for Autodock-Vina semi-flexible docking and found that the binding energies of COL1A1 with FA and (E,E)-3,5-Di-O-caffeoylquinic acid, COL1A2 with FA and Baicalin and MMP13 with Salvianolic acid, (E,E)-3,5-Di-O-caffeoylquinic acid and FA were ≤ − 5.0 kcal·mol^−1^, and the RMSD < 2.00. The core proteins were combined with the compounds predominantly through hydrogen bonding, and detailed information of the bonding sites is provided in Table 4. The above results demonstrated that the core proteins could form stable docking with compounds (Fig. 13B–H).

**Table 4:** The result of virtual screening

Protein (Binding Site)	Compound	Structure	DS(LibDockScore)	Vina(kcal·mol^−1^)	RMSD	Hydrogen bond interaction
COL1A1 (5K31)	Salvianolic acid		150.441	− 4.6	1.531	LYS:35, PRO:37, ARG:39, ASP:59, GLN:62
COL1A1 (5K31)	FA		149.345	− 5.3	1.651	ILE:27, ARG:28, LYS:35, ASP:59, GLY:63, ASN:65
COL1A1 (5K31)	(E,E)-3,5-Di-O-caffeoylquinic acid		143.163	− 5.2	1.841	ASN:26, ASN:36, PRO:37, ARG:39, GLY:62, GLY:63
COL1A2 (5CVA)	FA		125.939	− 5.7	1.785	ARG:37, ASP:41, GLN:42
COL1A2 (5CVA)	Baicalin		121.968	− 6.6	1.258	PRO:35, GLY:36, ARG:37, ASP:38
COL1A2 (5CVA)	Salvianolic acid		121.062	− 6.1	2.096	GLY:36, ARG:37, ASP:38, THR:40
MMP2 (7XJO)	Salvianolic acid		198.697	− 4.7	1.796	LEU:83, ALA:84, HIS:85, ALA:86,HIS:121,HIS:125
MMP2 (7XJO)	FA		167.272	− 4.9	2.079	VAL:118, HIS:131, ALA:140
MMP2 (7XJO)	(-)-Medicocarpin		145.696	− 4.9	1.815	ALA:86, HIS:121, GLU:130, HIS:131, TYR:143
MMP13 (2OW9)	Salvianolic acid		213.702	− 11.1	1.533	LEU:164, ALA:165, ALA:217, PHE:220, THR:224
MMP13 (2OW9)	(E,E)-3,5-Di-O-caffeoylquinic acid		209.107	− 11	0.985	PRO:215, ALA:217, PRO:221, THR:224, SER:229, HIS:230
MMP13 (2OW9)	FA		188.447	− 10.5	1.609	GLU:202, HIS:211, PRO:221, TYR:223, TYR:225, THR:226, MET:232

### Molecular dynamics simulation

**Fig.14:**
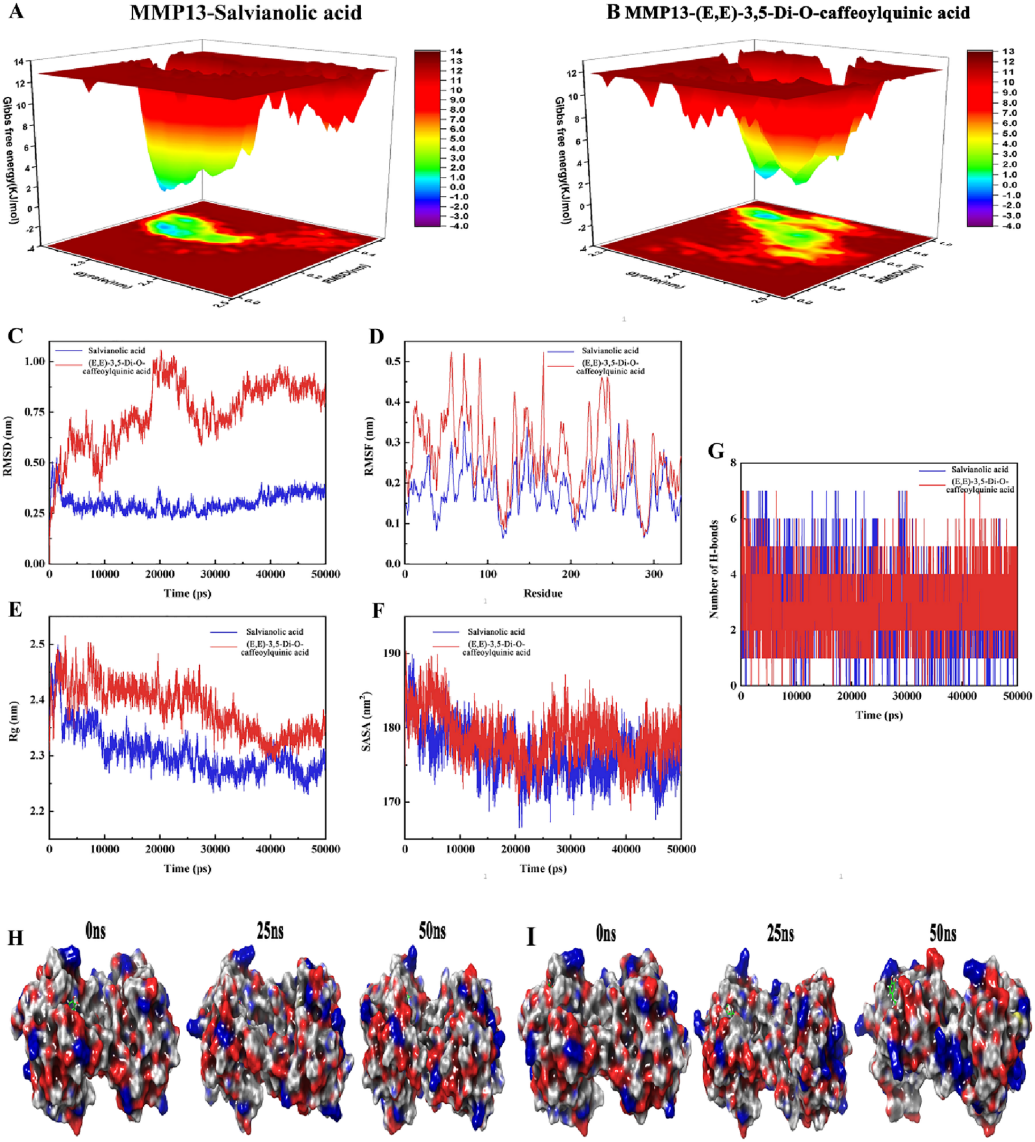
The results of MDS. 3D figures of free energy landscape of **A** MMP13-Salvianolic acid and **B** MMP13-(E,E)-3,5-Di-O-caffeoylquinic acid. **C** RMSD of backbone atoms of docked complex of Salvianolic acid (blue) and docked complex of (E,E)-3,5-Di-O-caffeoylquinic acid (red). **D** RMSF of the complex of Salvianolic acid (blue) and (E,E)-3,5-Di-O-caffeoylquinic acid (red). **E** Rg of complex of Salvianolic acid (blue) and (E,E)-3,5-Di-O-caffeoylquinic acid (red). **F** SASA of the complex of Salvianolic acid (blue) and (E,E)-3,5-Di-O-caffeoylquinic acid (red). **G** Hydrogen bonds interaction of the complex of Salvianolic acid (blue) and (E,E)-3,5-Di-O-caffeoylquinic acid (red). The conformational changes of the complexes of MMP13-Salvianolic acid **H** and MMP13-(E,E)-3,5-Di-O-caffeoylquinic acid **I** at 25 ns intervals in MDS

The free energy profiles results are shown in Fig. 14A, B, which revealed that the energy of the MMP13-Salvianolic acid complex fluctuated from − 2.0 to 14.0 kJ/mol and had only one free energy basin. In comparison, the energy of the MMP13-(E,E)-3,5-Di-O-caffeoylquinic acid complex fluctuated from − 2.0 to 13.0 kJ/mol and had two free energy basins with a highly stable energy distribution. RMSD is used to measure the stability of complexes, and the RMSD value of the MMP13-Salvianolic acid complex fluctuated at 0.25 nm, while the RMSD value of the MMP13-(E,E)-3,5-Di-O-caffeoylquinic acid complex fluctuated at 0.75 nm (Fig. 14C). RMSF is used to visualize the partial conformation of complexes during simulation (Fig. 14D). Both systems showed similar fluctuations at the same protein amino acid site. Next, the radius of gyration (Rg) is used to evaluate the structural compactness of complexes. The results showed that fluctuations of both complexes corresponded precisely to the trend of RMSD, and the MMP13-Salvianolic acid complex was less volatile than MMP13-(E,E)-3,5-Di-O-caffeoylquinic acid complex (Fig. 14E). In addition, SASA is used to assess protein solvent reachable surface variations, and it was found that the MMP13-Salvianolic acid complex exhibited a steady decline before reaching equilibrium, whereas the MMP13-(E,E)-3,5-Di-O-caffeoylquinic acid complex showed a falling-rising-falling fluctuation during the 50 ns simulation (Fig. 14F). The hydrogen bonding change curves for the two systems are shown in Fig. 14G. Finally, the conformational changes of the two complexes during the 50 ns MDS are shown in Fig. 14H, I. All these results showed that both MMP13-Salvianolic acid and MMP13-(E,E)-3,5-Di-O-caffeoylquinic acid complexes could reach a stable binding state during 50 ns MDS.

## Discussion

Knee osteoarthritis is characterised by joint pain and limited knee movement or even disability, which imposes a heavy burden on life [4]. Traditional Chinese Medicine (TCM) plays an essential function in treating KOA owing to multi-components, multi-targets, security and efficacy. In recent years, the clinical application of TBHLD has achieved excellent efficacy in early KOA patients, relieving painful symptoms and improving knee function remarkably. However, the components and mechanisms of TBHLD in treating KOA have not yet been explored. Therefore, this study aimed to explore the molecular mechanism of TBHLD in treating KOA based on bioinformatics analysis and experimental verification.

In this study, 206 bioactive components and 187 targets were screened from TBHLD. Besides, 50 intersecting genes were identified between TBHLD and KOA, and 20 core targets were calculated. They were enriched in ECM organization, extracellular structure organization, ECM-receptor interaction, PI3K-Akt and TNF pathways. It was found that the activation of PI3K-Akt pathway inhibited chondrocyte apoptosis and promoted chondrocyte proliferation [21, 22]. In addition, the ECM-receptor interaction pathway was a critical signaling and metabolic pathway in the progression of KOA [23]. The expression of type I collagens were enhanced in OA [24], and MMP2 and MMP13 were upregulated [25, 26], which disrupted the internal homeostasis of collagen, further accelerated ECM degradation and accelerated catabolism of KOA cartilage.

In the study, serum samples were collected from normal rats and TBHLD group. Qualitative and quantitative LC–MS analysis revealed the presence of 32 differentially expressed serum components in TBHLD-treated serum, elucidating the active ingredients and pharmacological mechanisms of TBHLD in the treatment of KOA. In addition, the OA chondrocyte models were successfully induced with IL-1β, and TBHLD markedly improved proliferation of OA chondrocytes and decreased the expression of Col1a1, Col1a2, Mmp2, Mmp13 in OA chondrocytes. It was found that Col1a1, Col1a2 were expressed at a low level in normal chondrocytes but highly expressed in OA, and dysregulated expression of type I collagen acts as a critical pathogenic role in OA [24, 27]. In addition, the expression of Mmp2/Mmp13 was enhanced in OA cartilage tissue, which accelerated the process of KOA cartilage degeneration by cleaving internal peptide bonds and degrading ECM components [28].

The cartilage structure of the knee joints were destroyed and the quantity of subchondral bones decreased markedly, and the trabecular arrangements were disturbed [29]. Micro-CT results showed that TBHLD significantly protected cartilage structure and ameliorated the loss of subchondral bone. In addition, the articular cartilage surface of OA was incomplete, cartilage layers were thinned or absent, and fissures were locally observed and penetrated deeply into the subchondral bone layer, and the subchondral bone structure were disturbed with lesion defects [30]. In addition, HE and SO&FG staining showed that TBHLD could promote the repair of cartilage lesions. The degradation of ECM by Mmp2 and Mmp13 was a major mechanism causing the degradation of KOA cartilage by pathological softening [26, 31]. Type II collagen protected articular cartilage is a major component of the cartilage ECM [32]. We found that TBHLD markedly decreased the expression of Mmp2 and Mmp13 proteins, and enhanced type II collagen expression, inhibited the degradation of OA cartilage and promoted the repair of OA cartilage lesions.

In the study, we found that salvianolic acid, folic acid, (E, E)-3,5-Di-O-caffeoylquinic acid, baicalin, (-)-medicocarpin in TBHLD could form a satble binding to Col1a1, Col1a2, Mmp2, Mmp13. Salvianolic acid inhibited the release of inflammatory cytokines from IL-1β-induced OA chondrocytes and had a protective effect against osteoarthritis [33] through inhibiting the activation of NF-κB and p38/MAPK pathways to exert anti-inflammatory effects in OA [34]. In addition, folic acid was found to protect chondrocytes through NF-κB/MAPK pathway and regulate the activity of macrophages [35]. Baicalin inhibited the expression of IL-1β-induced inflammatory cytokines by disrupting NF-κB pathway [36], and inhibited ECM degradation [37] and promoted ECM anabolism [38]. Therefore, they may be used as potential active components for targeting and regulating the expression of type I collagen and MMP2/13 to alleviate KOA cartilage degeneration.

This study demonstrates the innovative utilization of big data mining prediction and in vitro and in vivo experimental verification to substantiate the therapeutic effect of the traditional Chinese medicine formulation TBHLD on KOA. The comprehensive approach employed in this study involves the regulation of extracellular matrix synthesis and metabolism in chondrocytes through multiple components, targets, and pathways. The findings provide novel insights and molecular biological evidence for the prevention and treatment of KOA using traditional Chinese medicine and offer valuable guidance for its clinical application.

## Conclusions

In this study, we identified the components, DEGs and potential mechanism of TBHLD alleviating degeneration of KOA cartilage. We found that the core targets were enriched in ECM interaction pathways and the virtual screening for molecular docking and MDS results showed that the active components of TBHLD had steady binding conformations with core genes. The experimental validation has shown that TBHLD ameliorated the cartilage lesions and restored the cartilage abrasion. However, the active components of TBHLD and their molecular mechanisms should be explored in further experimental studies. In conclusion, TBHLD inhibited ECM degradation by regulating the expression of type I collagens and Mmps to ameliorate cartilage degeneration in KOA.

## Supplementary Information

**Additional file 1: Table S1.** the primer sequences. **Table S2.** the proliferative activity of OA chondrocytes. **Table S3.** the relative expression of core genes. **Table S4.** the change in bone mass in different groups. **Table S5.** the OARSI score in different groups. **Table S6.** the relative expression of Col II and Mmp2/Mmp13 proteins in different groups.

**Additional file 2:** The bioactive components and potential targets of TBHLD.

## Data Availability

The datasets used and analyzed during the current study are available from the corresponding author upon reasonable request.

## Abbreviations

BC: Betweenness centrality
BMD: Bone mineral density
BP: Biological process
BV/TV: Trabecular bone volume/tissue volume
CC: Closeness centrality
CC: Cellular component
DEGs: Differentially expression genes
DL: Drug-likeness
ECM: Extracellular matrix
GO: Gene ontology
IL-1β: Interleukin-1beta
KEGG: Kyoto encyclopedia of genes and genomes
KOA: Knee osteoarthritis
MDS: Molecular dynamics simulation
MF: Molecular function
MMPs: Matrix metalloproteinases
OA: Osteoarthritis
OARSI: Osteoarthritis research society international
OB: Oral Bioavailability
Po.tot: Total porosity
PPI: Protein–protein interaction
Tb.N: Trabecular number
Tb.Th: Trabecular thickness
