# Supplementary material for: Tongbi Huoluo Decoction alleviates cartilage degeneration in knee osteoarthritis by inhibiting degradation of extracellular matrix
Source: Chin Med. 2023 Jul 28;18:91. doi: 10.1186/s13020-023-00802-z (PMC10385923; doi:10.1186/s13020-023-00802-z)
Supplement: Supplementary file 1 — Additional file 1: Table S1. the primer sequences. Table S2. the proliferative activity of OA chondrocytes. Table S3. the relative expression of core genes. Table S4. the change in bone mass in different groups. Table S5. the OARSI score in different groups. Table S6. the relative expression of Col II and Mmp2/Mmp13 proteins in different groups. [file 13020_2023_802_MOESM1_ESM.doc]

| **Genes** | **Towards** | **Sequence (5'to 3')** |
| --- | --- | --- |
| **Gapdh** | **Forward** | **GCACCGTCAAGGCTGAGAAC** |
|  | **Reverse** | **TGGTGAAGACGCCAGTGGA** |
| **Mmp2** | **Forward** | **ATTGTATTTGATGGCATCGCTC** |
|  | **Reverse** | **ATTCATTCCCTGCAAAGAACAC** |
| **Ccnd1** | **Forward** | **GTCCTACTTCAAATGTGTGCAG** |
|  | **Reverse** | **GGGATGGTCTCCTTCATCTTAG** |
| **Col1a1** | **Forward** | **AAAGATGGACTCAACGGTCTC** |
|  | **Reverse** | **CATCGTGAGCCTTCTCTTGAG** |
| **Col1a2** | **Forward** | **CTCCATGGTGAGTTTGGTCTC** |
|  | **Reverse** | **CTTCCAATAGGACCAGTAGGAC** |
| **Col3a1** | **Forward** | **CTCAGGGTGTCAAGGGTGAAAGTG** |
|  | **Reverse** | **TGTACCAGCCAGACCAGGAAGAC** |
| **Runx2** | **Forward** | **AGGCAGTTCCCAAGCATTTCATCC** |
|  | **Reverse** | **TGGCAGGTAGGTGTGGTAGTGAG** |
| **Col5a1** | **Forward** | **CGTATGATGACCTCACCTATGG** |
|  | **Reverse** | **CGTAGTAGTTCTCGTCAAGGTT** |
| **Mmm13** | **Forward** | **CACTTTATGCTTCCTGATGACG** |
|  | **Reverse** | **TCTGGCGTTTTTGGATGTTTAG** |
| **Thbs2** | **Forward** | **AACAGCCCTGAGCCTCAGTA** |
|  | **Reverse** | **GAAGCAGGGGTTGGATAAACAG** |

Additional materials

**Table.1** the primer sequences

**Table.2 the proliferative activity of OA chondrocytes**

| **Time** | **Group** | **Number** | **Proliferative activity** | **Shapiro-Wilk**  **test** | ***F*** | ***P*** |
| --- | --- | --- | --- | --- | --- | --- |
| **24h** | **Con** | **6** | **1.000±0.039** | **0.653** | **102.201** | **0.000** |
|  | **OA^****^** | **6** | **0.730±0.016** | **0.656** |  |  |
|  | **0.625%^##^** | **6** | **0.790±0.017** | **0.833** |  |  |
|  | **1.25%^####^** | **6** | **0.815±0.022** | **0.567** |  |  |
|  | **2.50%^####^** | **6** | **0.833±0.024** | **0.212** |  |  |
|  | **5.00%** | **6** | **0.743±0.012** | **0.431** |  |  |
|  | **10.00%** | **6** | **0.705±0.026** | **0.361** |  |  |
| **48h** | **Con** | **6** | **1.000±0.052** | **0.926** | **36.239** | **0.000** |
|  | **OA^****^** | **6** | **0.760±0.023** | **0.402** |  |  |
|  | **0.625%** | **6** | **0.804±0.021** | **0.852** |  |  |
|  | **1.25%^#^** | **6** | **0.814±0.016** | **0.793** |  |  |
|  | **2.50%^####^** | **6** | **0.894±0.015** | **0.849** |  |  |
|  | **5.00%^###^** | **6** | **0.855±0.001** | **0.097** |  |  |
|  | **10.00%** | **6** | **0.734±0.007** | **0.228** |  |  |
| **72h** | **Con** | **6** | **1.000±0.042** | **0.614** | **462.969** | **0.000** |
|  | **OA^****^** | **6** | **0.728±0.031** | **0.109** |  |  |
|  | **0.625%^#^** | **6** | **0.804±0.012** | **0.556** |  |  |
|  | **1.25%^##^** | **6** | **0.835±0.014** | **0.188** |  |  |
|  | **2.50%^####^** | **6** | **0.882±0.028** | **0.311** |  |  |
|  | **5.00%^##^** | **6** | **0.825±0.031** | **0.936** |  |  |
|  | **10.00%^##^** | **6** | **0.626±0.003** | **0.375** |  |  |

****p < 0.0001 vs. Control; ##p < 0.01 , ###p < 0.001 and ####p < 0.0001vs. OA.

**Table.3** the relative expression of core genes

| **Genes** | **Group** | **Number** | **Relative expression** | **Shapiro-Wilk**  **test** | ***F*** | ***P*** |
| --- | --- | --- | --- | --- | --- | --- |
| **COL1A1** | **Con** | **3** | **1.050±0.083** | **0.057** | **177.601** | **0.000** |
|  | **OA^**^** | **3** | **4.754±0.356** | **0.097** |  |  |
|  | **1.25%^##^** | **3** | **0.086±0.008** | **0.127** |  |  |
|  | **2.50%^##^** | **3** | **0.042±0.004** | **0.969** |  |  |
|  | **5.00%^##^** | **3** | **0.047±0.013** | **0.341** |  |  |
| **COL1A2** | **Con** | **3** | **1.098±0.088** | **0.514** | **91.097** | **0.000** |
|  | **OA^**^** | **3** | **1.696±0.047** | **0.055** |  |  |
|  | **1.25%^####^** | **3** | **2.593±0.233** | **0.374** |  |  |
|  | **2.50%^####^** | **3** | **0.843±0.181** | **0.824** |  |  |
|  | **5.00%^####^** | **3** | **0.456±0.132** | **0.681** |  |  |
| **COL3A1** | **Con** | **3** | **0.933±0.083** | **0.470** | **61.336** | **0.000** |
|  | **OA^***^** | **3** | **1.713±0.222** | **0.449** |  |  |
|  | **1.25%** | **3** | **1.746±0.194** | **0.969** |  |  |
|  | **2.50%^####^** | **3** | **0.470±0.046** | **0.764** |  |  |
|  | **5.00%^####^** | **3** | **0.417±0.081** | **0.698** |  |  |
| **COL5A1** | **Con** | **3** | **1.204±0.223** | **0.742** | **113.432** | **0.000** |
|  | **OA^****^** | **3** | **2.745±0.149** | **0.074** |  |  |
|  | **1.25%** | **3** | **2.881±0.184** | **0.419** |  |  |
|  | **2.50%^####^** | **3** | **0.691±0.206** | **0.572** |  |  |
|  | **5.00%^####^** | **3** | **0.627±0.113** | **0.335** |  |  |
| **MMP2** | **Con** | **3** | **0.867±0.127** | **0.820** | **8.2** | **0.003** |
|  | **OA^*^** | **3** | **1.339±0.180** | **0.386** |  |  |
|  | **1.25%^#^** | **3** | **0.872±0.089** | **0.870** |  |  |
|  | **2.50%^##^** | **3** | **0.765±0.172** | **0.666** |  |  |
|  | **5.00%^##^** | **3** | **0.728±0.157** | **0.491** |  |  |
| **MMP3** | **Con** | **3** | **0.868±0.146** | **0.715** | **72.233** | **0.001** |
|  | **OA^*^** | **3** | **2.203±0.009** | **0.166** |  |  |
|  | **1.25%** | **3** | **1.942±0.292** | **0.276** |  |  |
|  | **2.50%** | **3** | **1.845±0.114** | **0.430** |  |  |
|  | **5.00%** | **3** | **5.139±0.414** | **0.118** |  |  |
| **MMP13** | **Con** | **3** | **0.982±0.103** | **0.705** | **53.433** | **0.000** |
|  | **OA^*^** | **3** | **35.719±4.032** | **0.966** |  |  |
|  | **1.25%^#^** | **3** | **2.026±0.281** | **0.214** |  |  |
|  | **2.50%^#^** | **3** | **1.641±0.130** | **0.772** |  |  |
|  | **5.00%^#^** | **3** | **1.609±0.111** | **0.733** |  |  |
| **RUNX2** | **Con** | **3** | **0.891±0.111** | **0.938** | **1144.219** | **0.000** |
|  | **OA^**^** | **3** | **17.398±1.280** | **0.732** |  |  |
|  | **1.25%** | **3** | **12.988±1.075** | **0.293** |  |  |
|  | **2.50%^#^** | **3** | **7.768±0.302** | **0.647** |  |  |
|  | **5.00%^#^** | **3** | **8.505±0.137** | **0.920** |  |  |
| **THBS2** | **Con** | **3** | **1.223±0.252** | **0.666** | **160.474** | **0.000** |
|  | **OA^****^** | **3** | **3.339±0.131** | **0.631** |  |  |
|  | **1.25%^####^** | **3** | **1.748±0.172** | **0.703** |  |  |
|  | **2.50%^####^** | **3** | **0.809±0.015** | **0.363** |  |  |
|  | **5.00%^####^** | **3** | **0.523±0.072** | **0.884** |  |  |
| **CCND1** | **Con** | **3** | **0.940±0.084** | **0.263** | **163.81** | **0.000** |
|  | **OA** | **3** | **1.823±0.283** | **0.491** |  |  |
|  | **1.25%** | **3** | **1.508±0.091** | **0.495** |  |  |
|  | **2.50%^#^** | **3** | **0.273±0.028** | **0.558** |  |  |
|  | **5.00%^#^** | **3** | **0.168±0.016** | **0.872** |  |  |

*p < 0.05 , **p < 0.01 , ***p < 0.001 and****p < 0.0001 vs. Control; ^#^p < 0.05 , ^##^p < 0.01 and ^####^p < 0.0001vs. OA.

**Table.4** the change in bone mass in different groups

| **Indexs** | **Group** | **Number** | **Values** | **Shapiro-Wilk**  **test** | ***F*** | ***P*** |
| --- | --- | --- | --- | --- | --- | --- |
| **BMD** | **Sham** | **6** | **0.580±0.024** | **0.270** | **9.319** | **0.000** |
|  | **Model^*^** | **6** | **0.517±0.040** | **0.081** |  |  |
|  | **TBHLD.Low^##^** | **6** | **0.607±0.031** | **0.982** |  |  |
|  | **TBHLD.High** | **6** | **0.519±0.045** | **0.672** |  |  |
| **BV/TV** | **Sham** | **6** | **36.573±3.190** | **0.431** | **9.117** | **0.001** |
|  | **Model** | **6** | **30.281±3.723** | **0.101** |  |  |
|  | **TBHLD.Low^###^** | **6** | **40.674±3.774** | **0.932** |  |  |
|  | **TBHLD.High** | **6** | **31.532±4.696** | **0.721** |  |  |
| **Po（tot）** | **Sham** | **6** | **63.427±3.190** | **0.431** | **9.117** | **0.001** |
|  | **Model** | **6** | **69.719±3.723** | **0.101** |  |  |
|  | **TBHLD.Low^###^** | **6** | **59.326±3.774** | **0.932** |  |  |
|  | **TBHLD.High** | **6** | **68.468±4.696** | **0.721** |  |  |
| **Tb.N** | **Sham** | **6** | **3.609±0.235** | **0.113** | **9.201** | **0.000** |
|  | **Model** | **6** | **3.256±0.418** | **0.739** |  |  |
|  | **TBHLD.Low^###^** | **6** | **4.690±0.081** | **0.917** |  |  |
|  | **TBHLD.High** | **6** | **3.334±0.438** | **0.162** |  |  |
| **Tb.Th** | **Sham** | **6** | **0.102±0.003** | **0.515** | **9.964** | **0.045** |
|  | **Model** | **6** | **0.093±0.009** | **0.063** |  |  |
|  | **TBHLD.Low** | **6** | **0.094±0.007** | **0.991** |  |  |
|  | **TBHLD.High** | **6** | **0.095±0.008** | **0.991** |  |  |

*p < 0.05 vs. Sham; ^##^p < 0.01 and ^###^p < 0.001vs. Model.

**Table.5** the OARSI score in different groups

| **Group** | **Sham** | **Model** | **TBHLD.Low** | **TBHLD.High** |
| --- | --- | --- | --- | --- |
| **OARSI(`x±s)** | **0.500±0.447** | **4.833±0.753^****^** | **1.167±0.683^####^** | **2.333±0.817^####^** |
| **Shapiro-Wilk**  **test** | **0.167** | **0.212** | **0.093** | **0.091** |
| ***F*** | **45.936** | | | |
| ***P*** | **0.000** | | | |

****p < 0.0001 vs. Sham; ^####^p < 0.0001vs. Model.

**Table.6** the relative expression of Col II and Mmp2/Mmp13 proteins in different groups

| **Proteins** | **Group** | **Number** | **Relative expression** | **Shapiro-Wilk**  **test** | ***F*** | ***P*** |
| --- | --- | --- | --- | --- | --- | --- |
| **Mmp2** | **Sham** | **3** | **6.220±1.013** | **0.208** | **20.957** | **0.000** |
|  | **Model^***^** | **3** | **11.519±1.057** | **0.161** |  |  |
|  | **TBHLD.Low^###^** | **3** | **5.652±1.218** | **0.979** |  |  |
|  | **TBHLD.High^#^** | **3** | **8.584±0.678** | **0.893** |  |  |
| **Col II** | **Sham** | **3** | **28.310±2.058** | **0.797** | **60.570** | **0.000** |
|  | **Model^****^** | **3** | **19.232±0.251** | **0.196** |  |  |
|  | **TBHLD.Low^##^** | **3** | **24.434±1.121** | **0.823** |  |  |
|  | **TBHLD.High** | **3** | **16.433±0.123** | **0.078** |  |  |
| **Mmp13** | **Sham** | **3** | **8.211±0.179** | **0.240** | **22.400** | **0.008** |
|  | **Model^*^** | **3** | **13.206±0.969** | **0.316** |  |  |
|  | **TBHLD.Low^#^** | **3** | **7.922±0.440** | **0.208** |  |  |
|  | **TBHLD.High** | **3** | **10.874±1.300** | **0.280** |  |  |

*p < 0.05 , ***p < 0.001 and ****p < 0.0001 vs. Sham; ^#^p < 0.05 , ^##^p < 0.01 and ^###^p < 0.001 vs. Model.
